# Supplementary figures and images for: Conditioned medium from M2b macrophages modulates the proliferation, migration, and apoptosis of pulmonary artery smooth muscle cells by deregulating the PI3K/Akt/FoxO3a pathway
Source: PeerJ. 2020 May 5;8:e9110. doi: 10.7717/peerj.9110 (PMC7207208; doi:10.7717/peerj.9110)

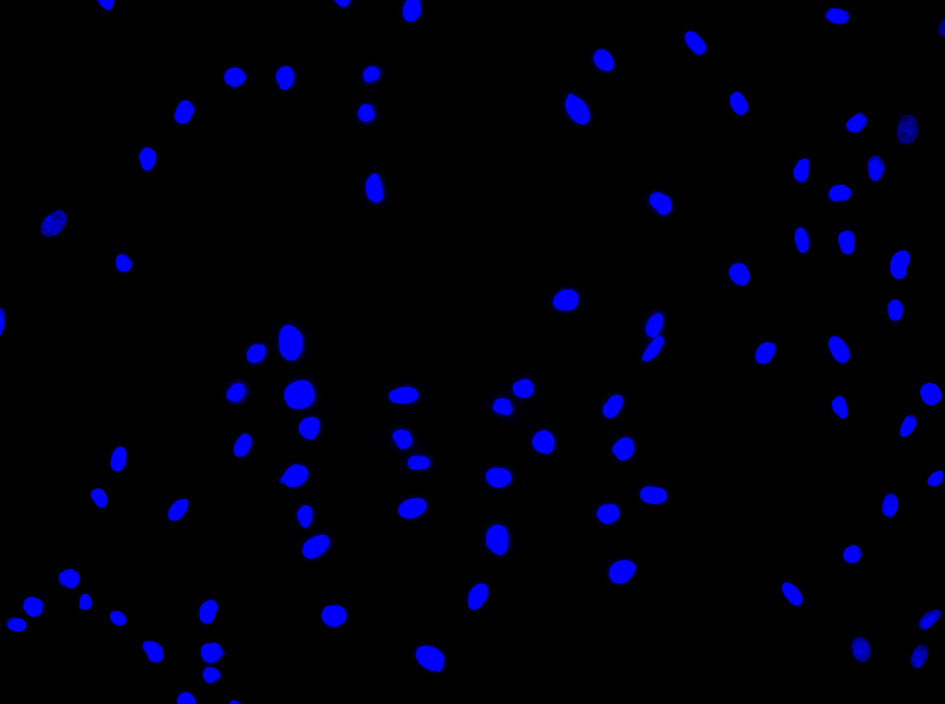

Supplement: Data S1 [file peerj-08-9110-s001.zip › Raw data-Figure 1A.png]

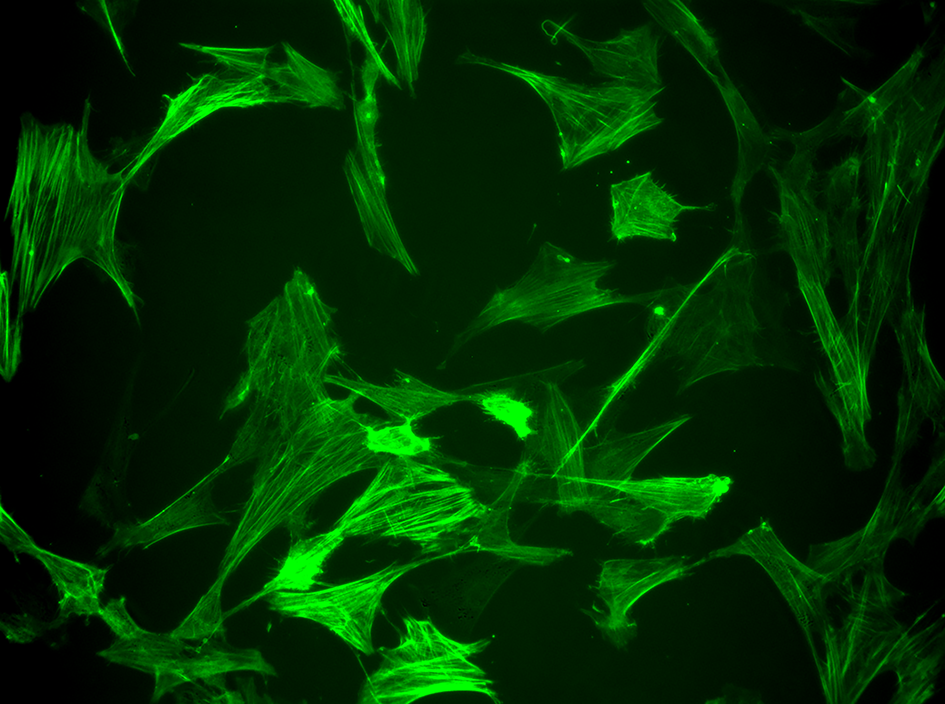

Supplement: Data S1 [file peerj-08-9110-s001.zip › Raw data-Figure 1B.png]

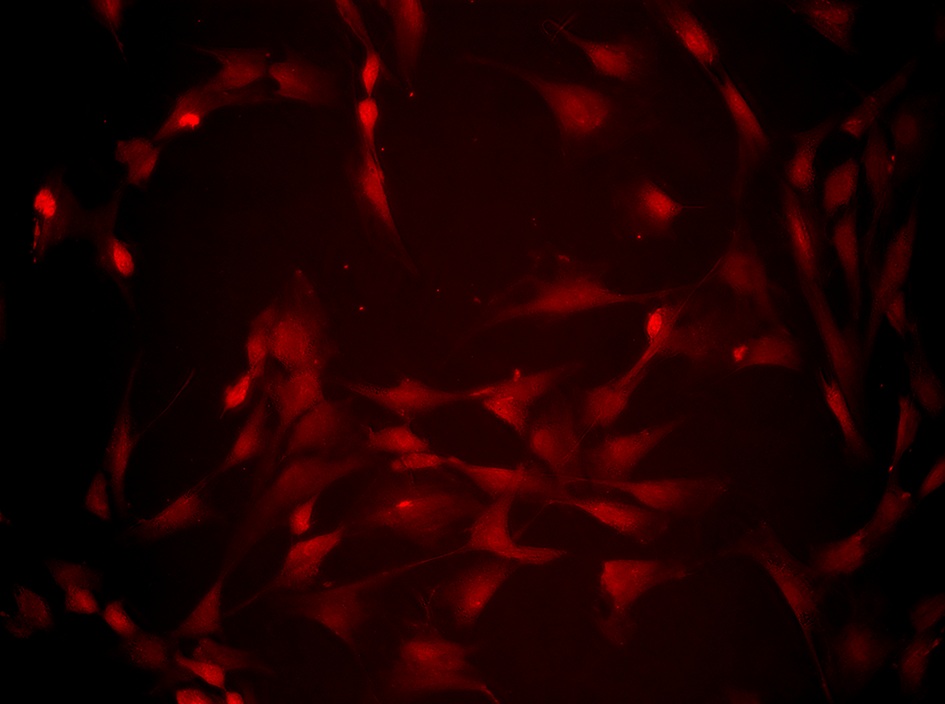

Supplement: Data S1 [file peerj-08-9110-s001.zip › Raw data-Figure 1C.png]

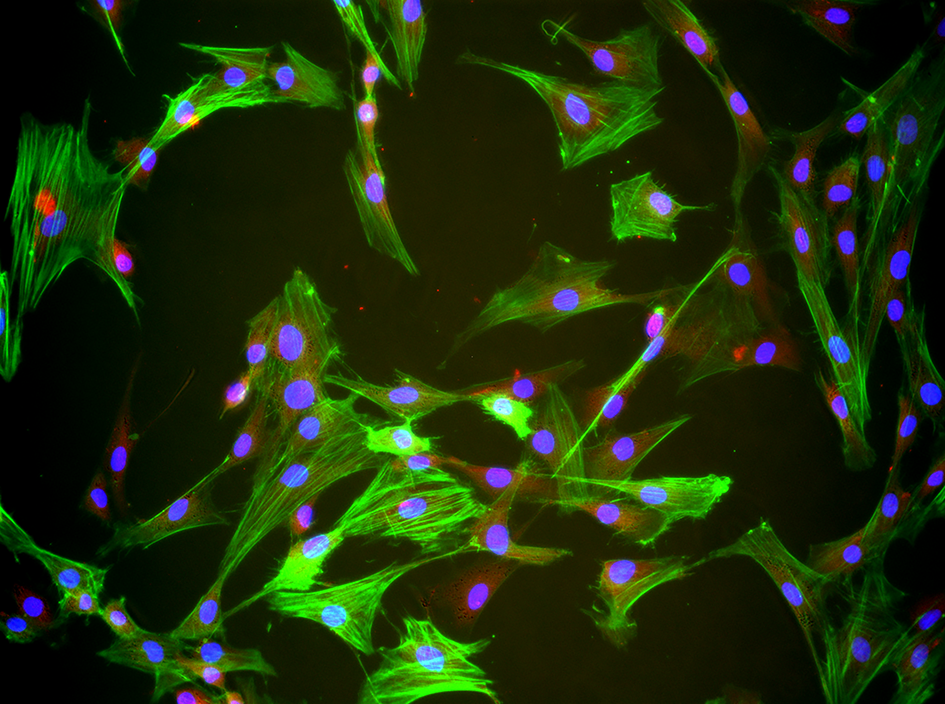

Supplement: Data S1 [file peerj-08-9110-s001.zip › Raw data-Figure 1D.png]

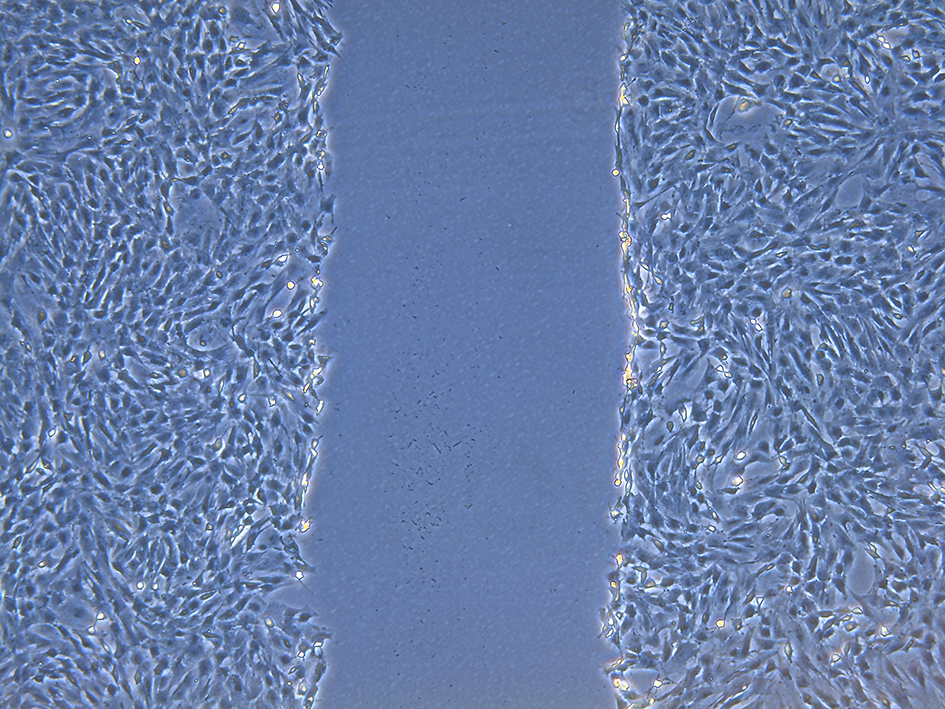

Supplement: Data S1 [file peerj-08-9110-s001.zip › Raw data-Figure 2B.png]

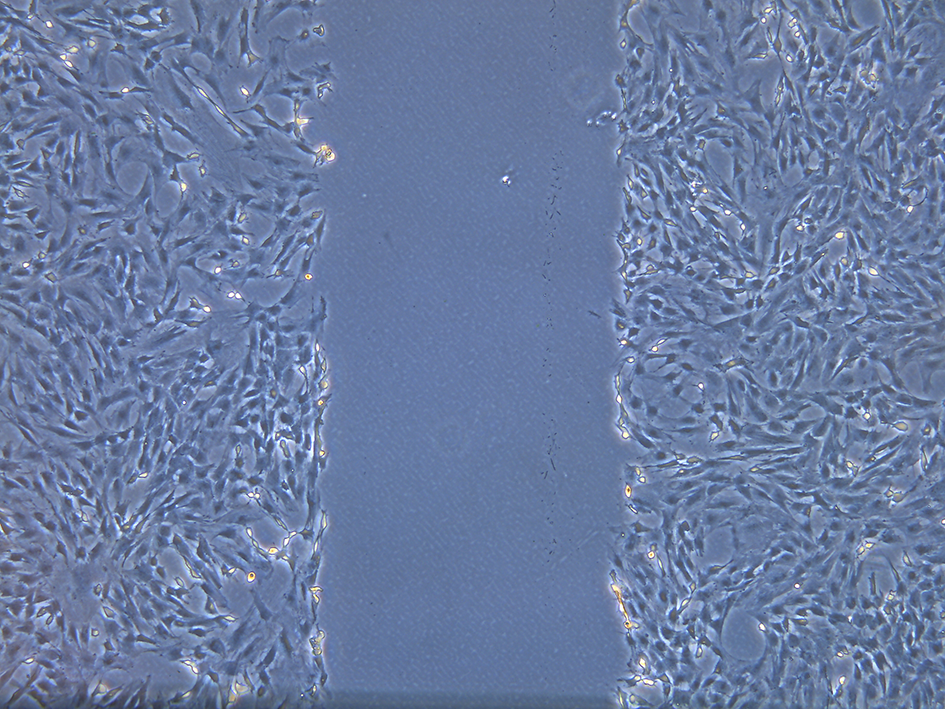

Supplement: Data S1 [file peerj-08-9110-s001.zip › Raw data-Figure 2C.png]

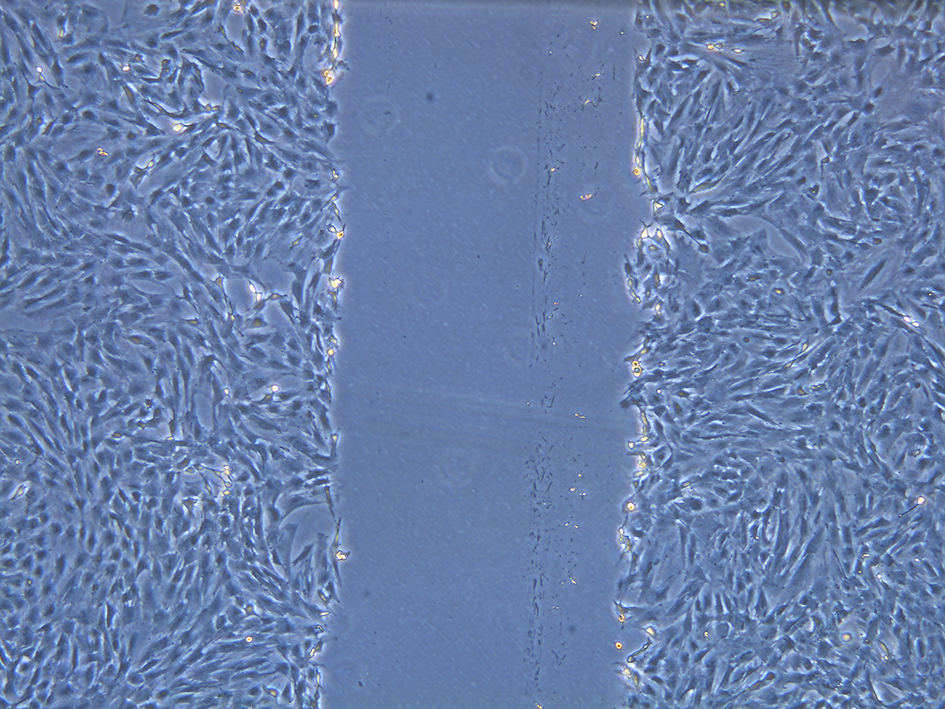

Supplement: Data S1 [file peerj-08-9110-s001.zip › Raw data-Figure 2D.png]

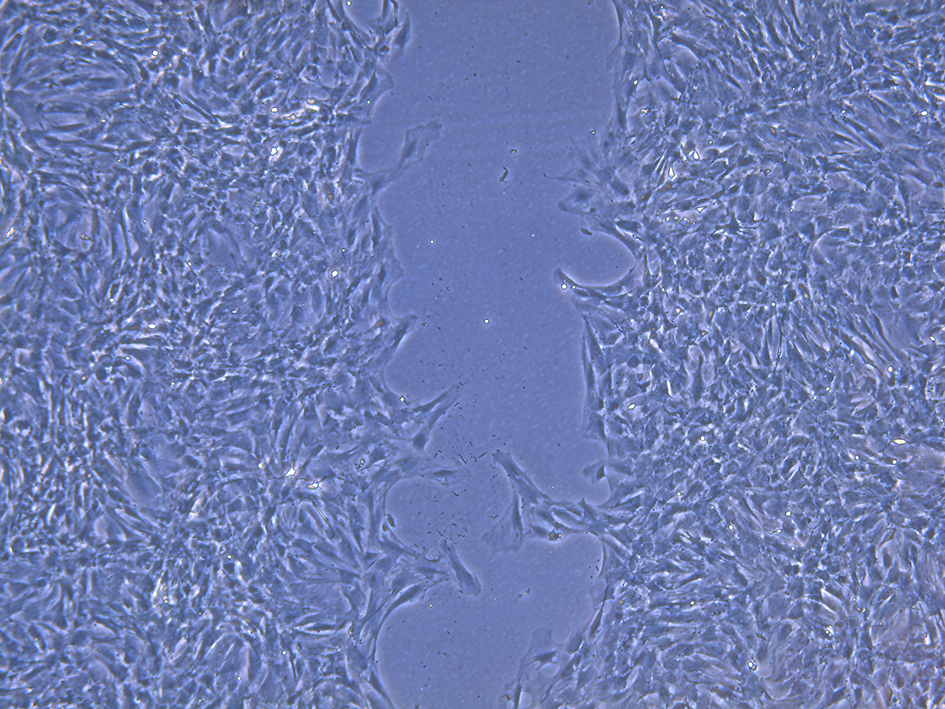

Supplement: Data S1 [file peerj-08-9110-s001.zip › Raw data-Figure 2E.png]

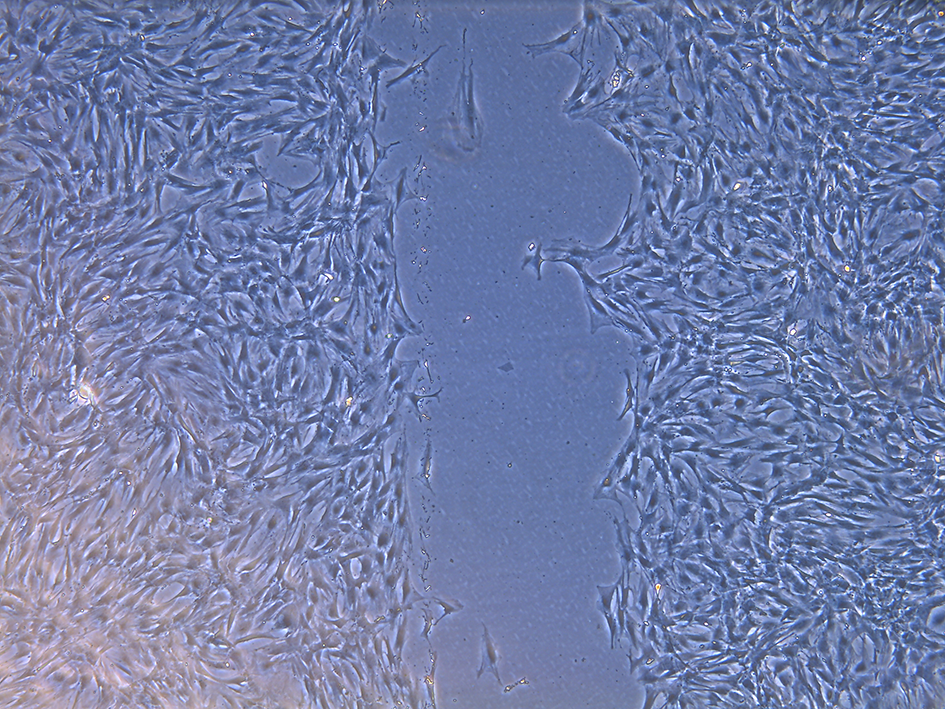

Supplement: Data S1 [file peerj-08-9110-s001.zip › Raw data-Figure 2F.png]

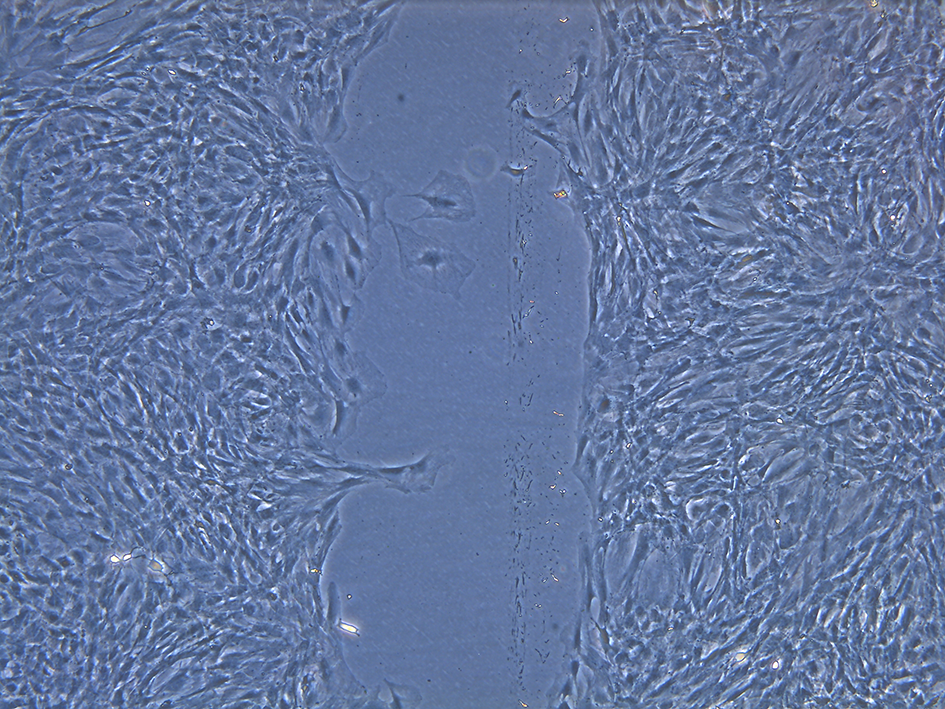

Supplement: Data S1 [file peerj-08-9110-s001.zip › Raw data-Figure 2G.png]

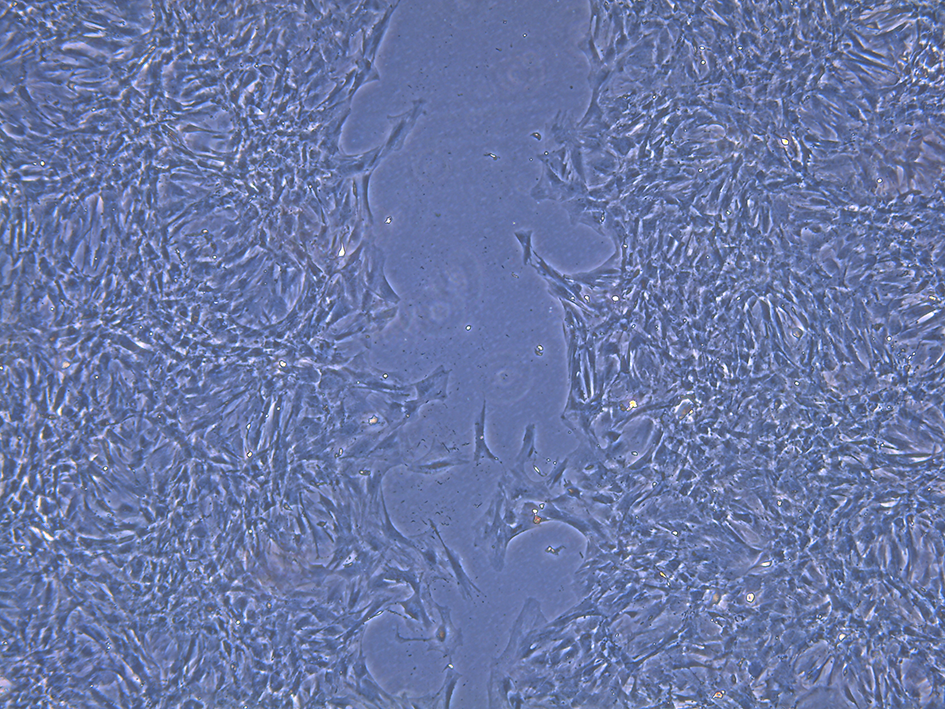

Supplement: Data S1 [file peerj-08-9110-s001.zip › Raw data-Figure 2H.png]

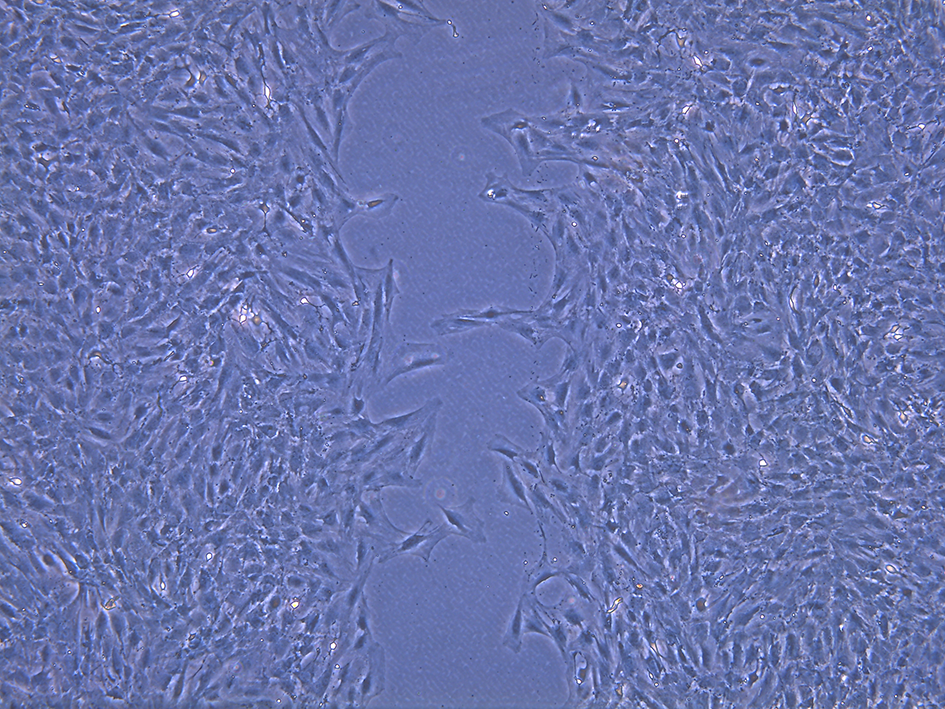

Supplement: Data S1 [file peerj-08-9110-s001.zip › Raw data-Figure 2I.png]

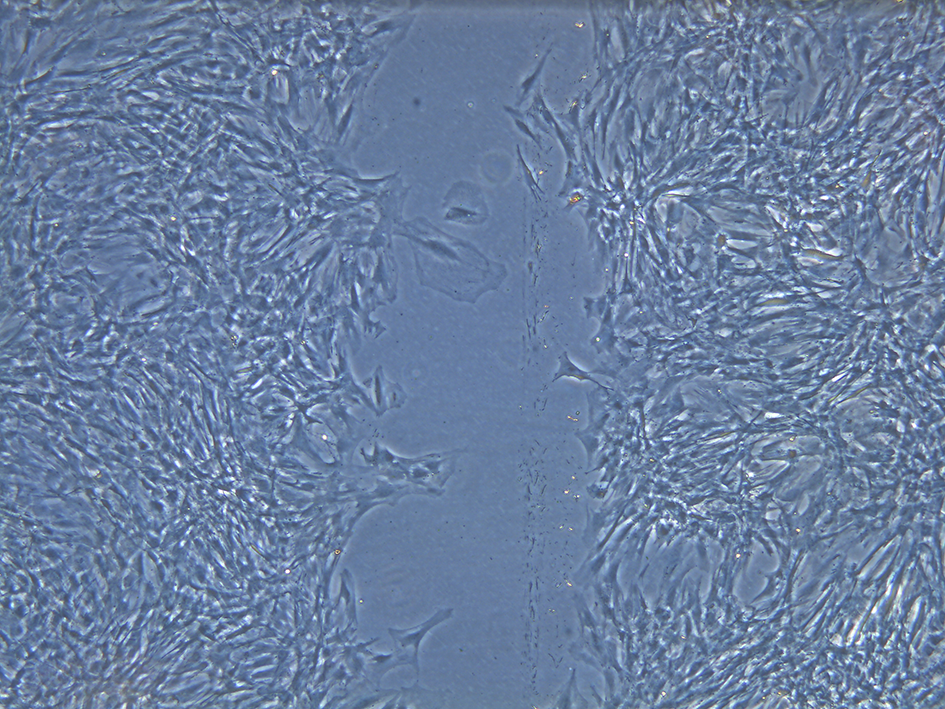

Supplement: Data S1 [file peerj-08-9110-s001.zip › Raw data-Figure 2J.png]

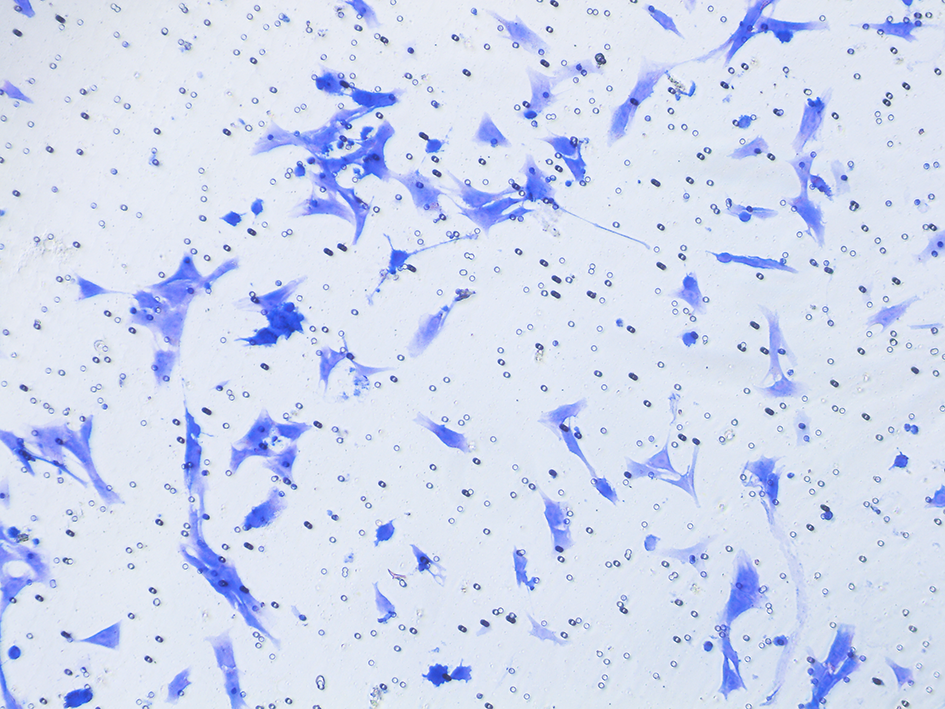

Supplement: Data S1 [file peerj-08-9110-s001.zip › Raw data-Figure 2L.png]

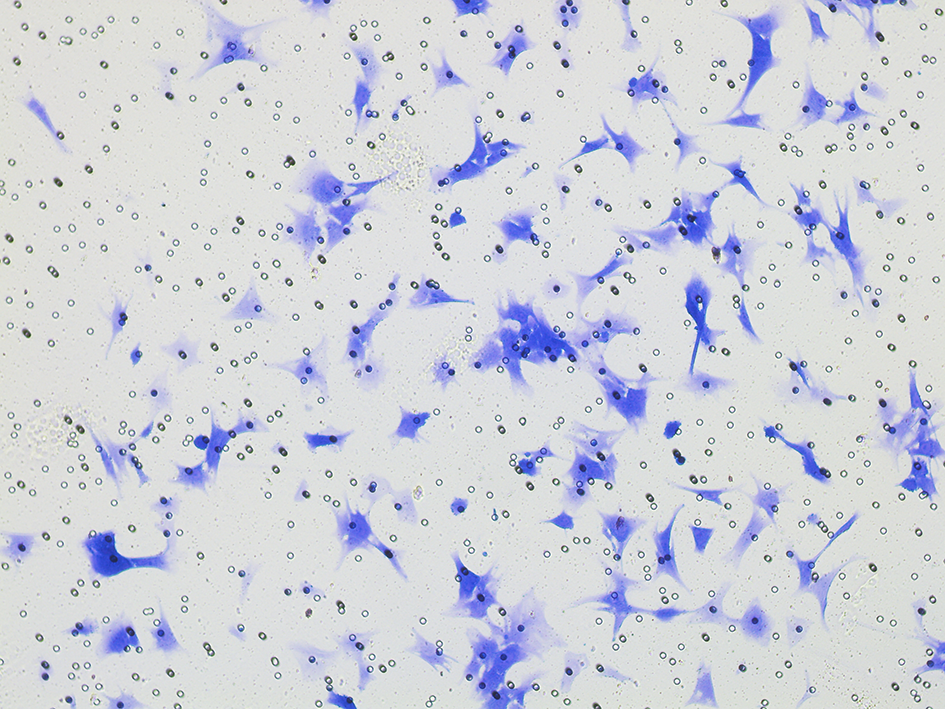

Supplement: Data S1 [file peerj-08-9110-s001.zip › Raw data-Figure 2M.png]

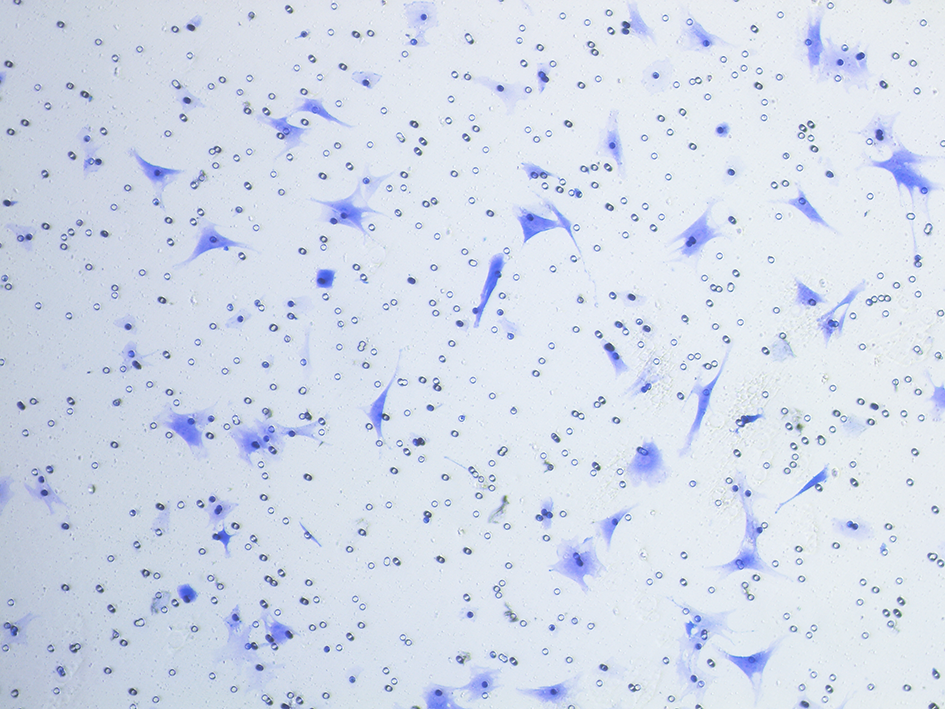

Supplement: Data S1 [file peerj-08-9110-s001.zip › Raw data-Figure 2N.png]

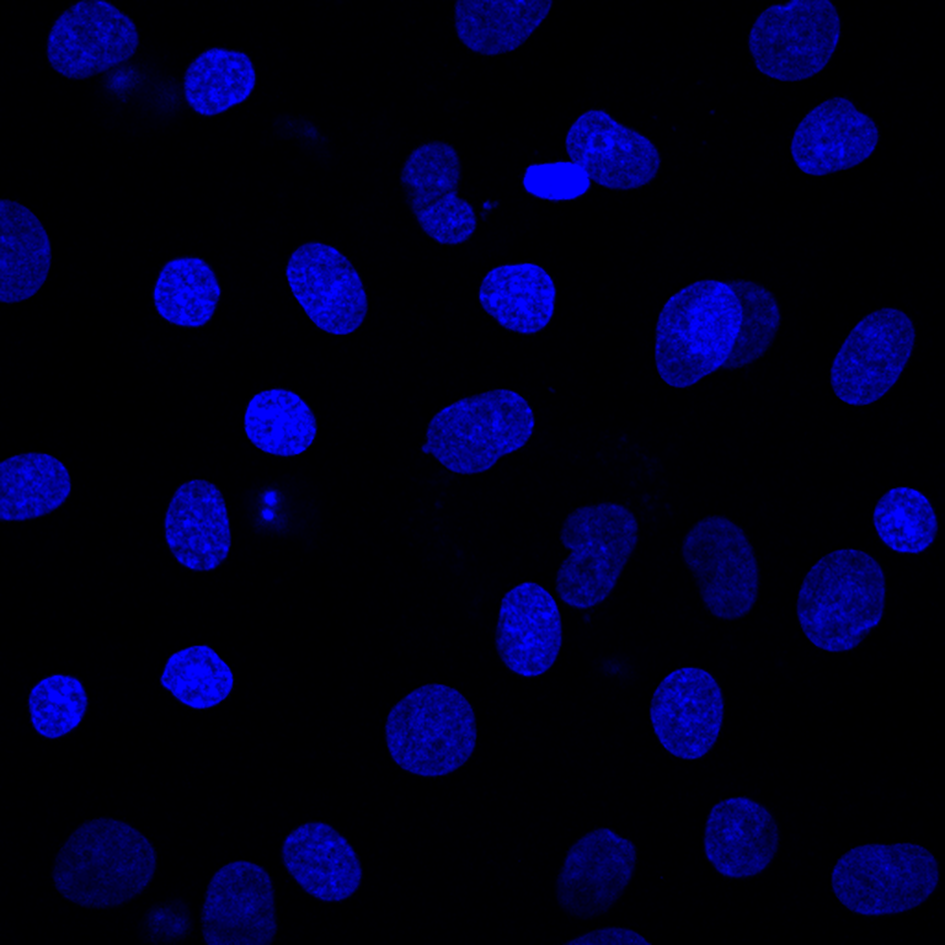

Supplement: Data S1 [file peerj-08-9110-s001.zip › Raw data-Figure 3A.png]

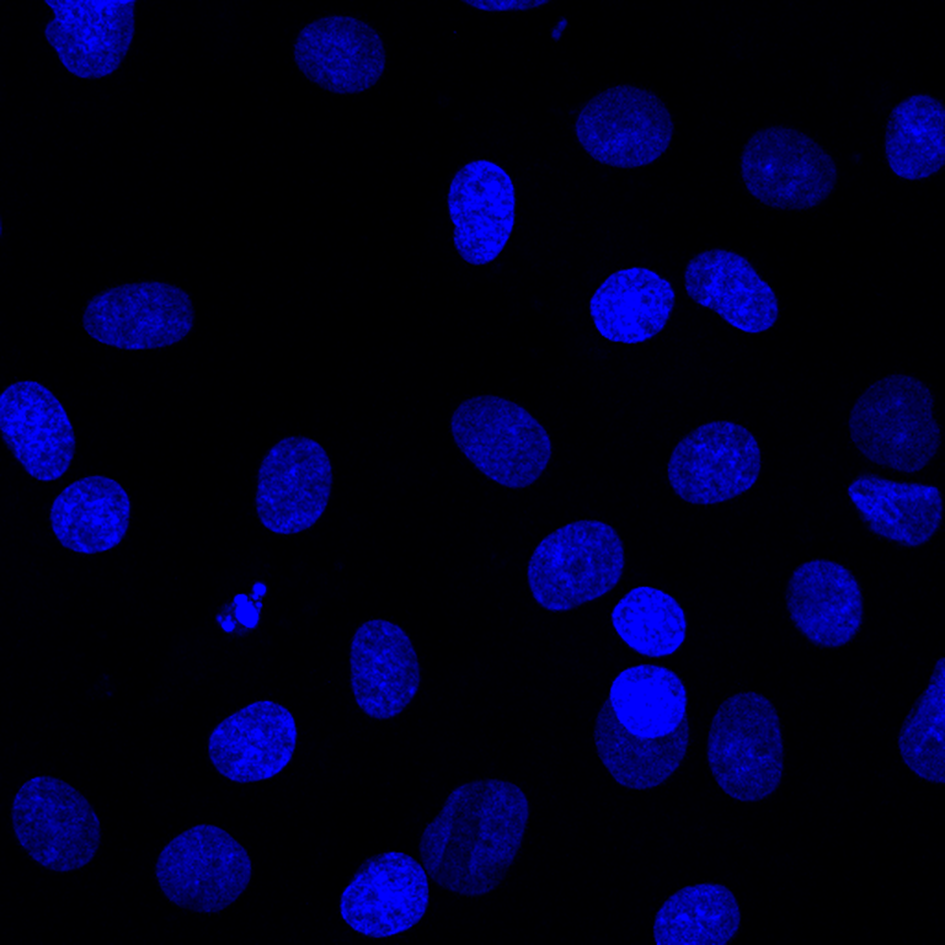

Supplement: Data S1 [file peerj-08-9110-s001.zip › Raw data-Figure 3B.png]

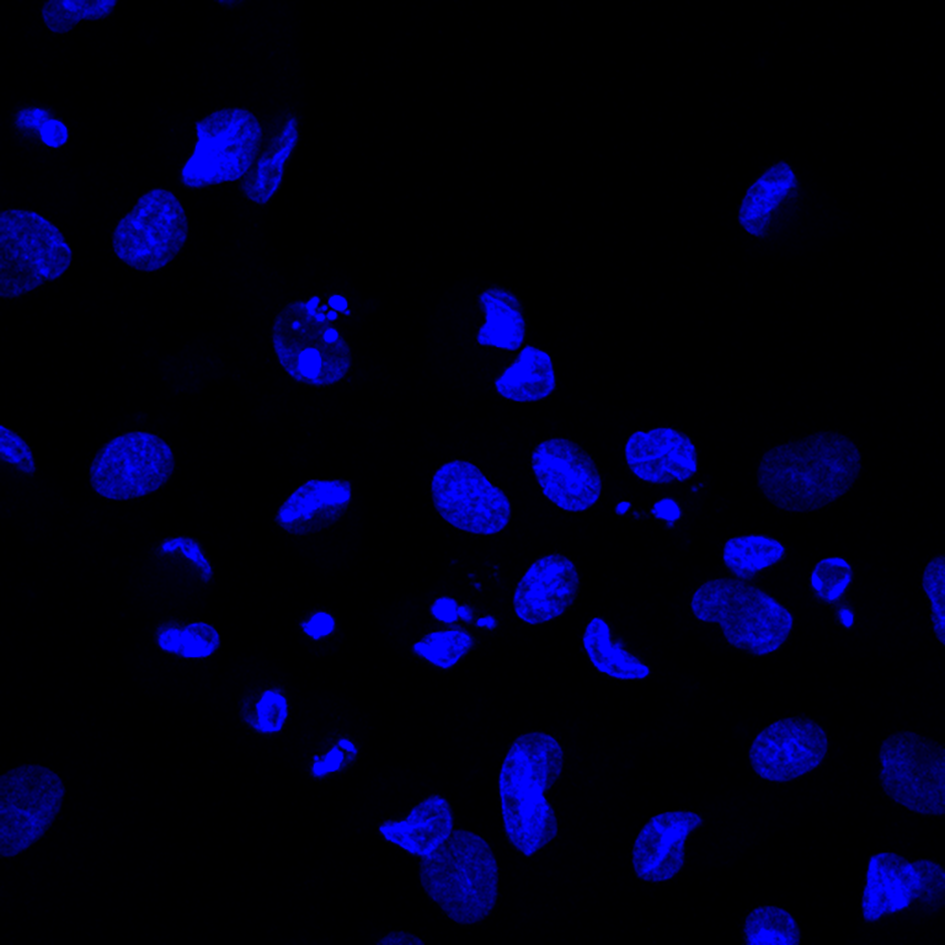

Supplement: Data S1 [file peerj-08-9110-s001.zip › Raw data-Figure 3C.png]

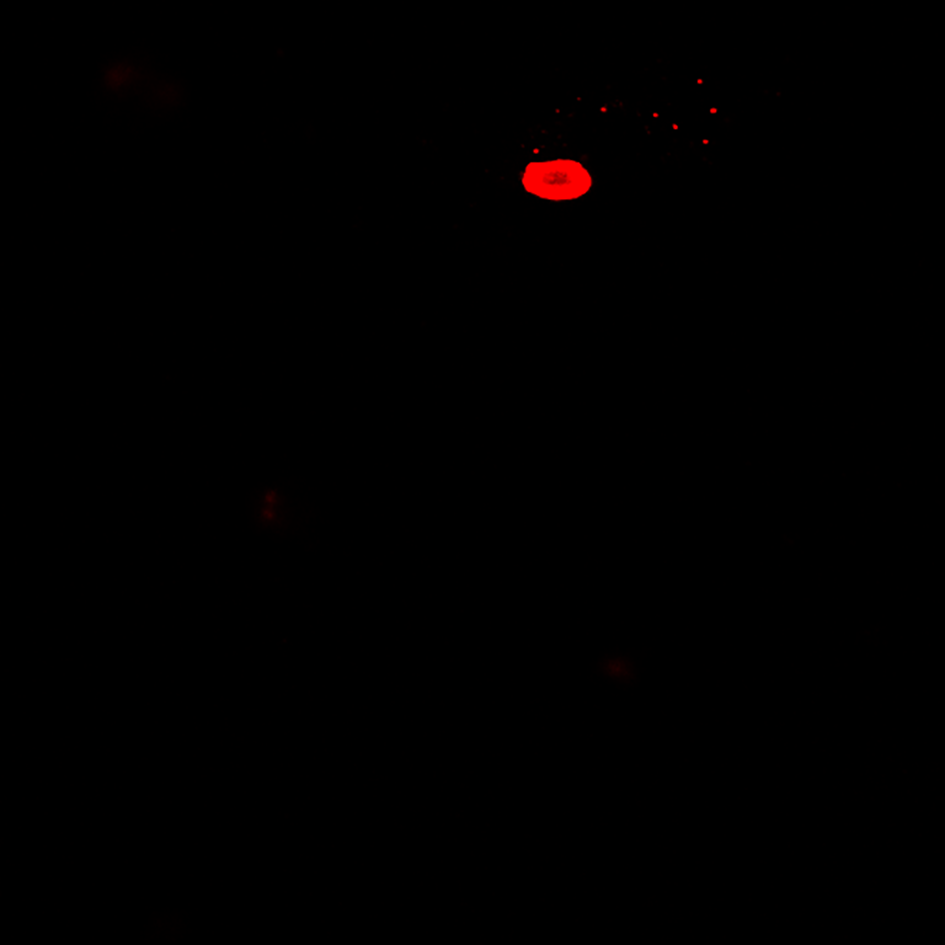

Supplement: Data S1 [file peerj-08-9110-s001.zip › Raw data-Figure 3D.png]

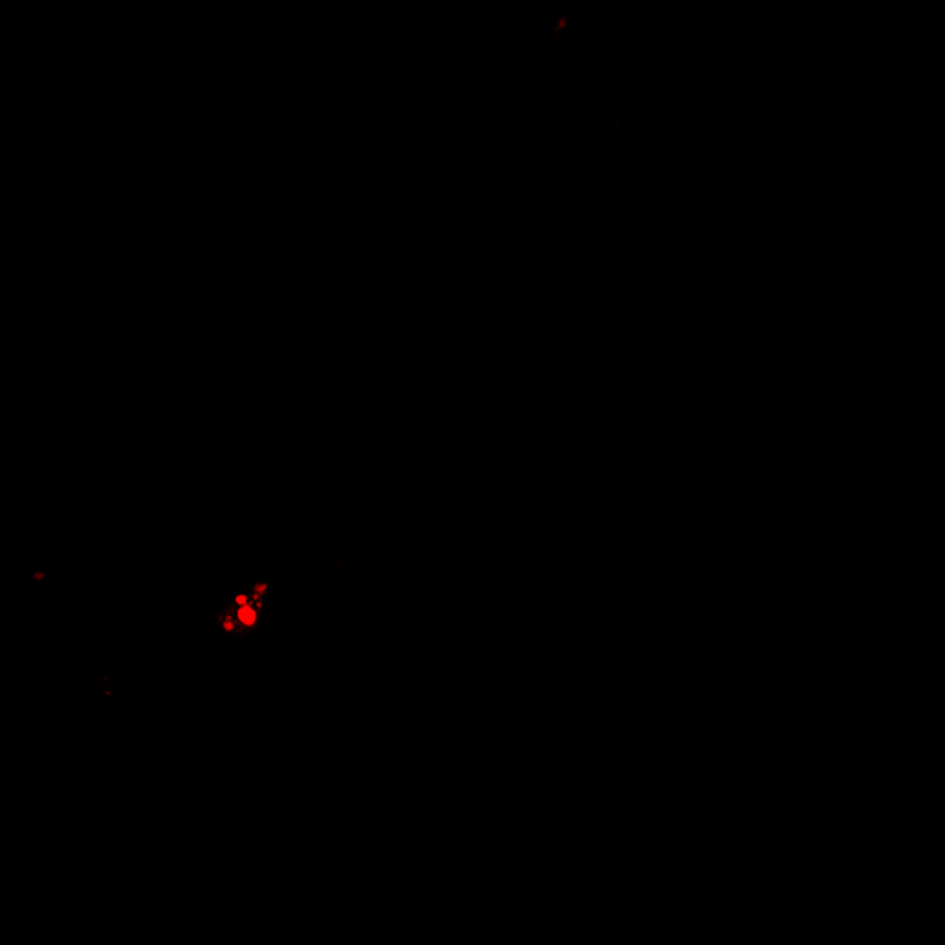

Supplement: Data S1 [file peerj-08-9110-s001.zip › Raw data-Figure 3E.png]

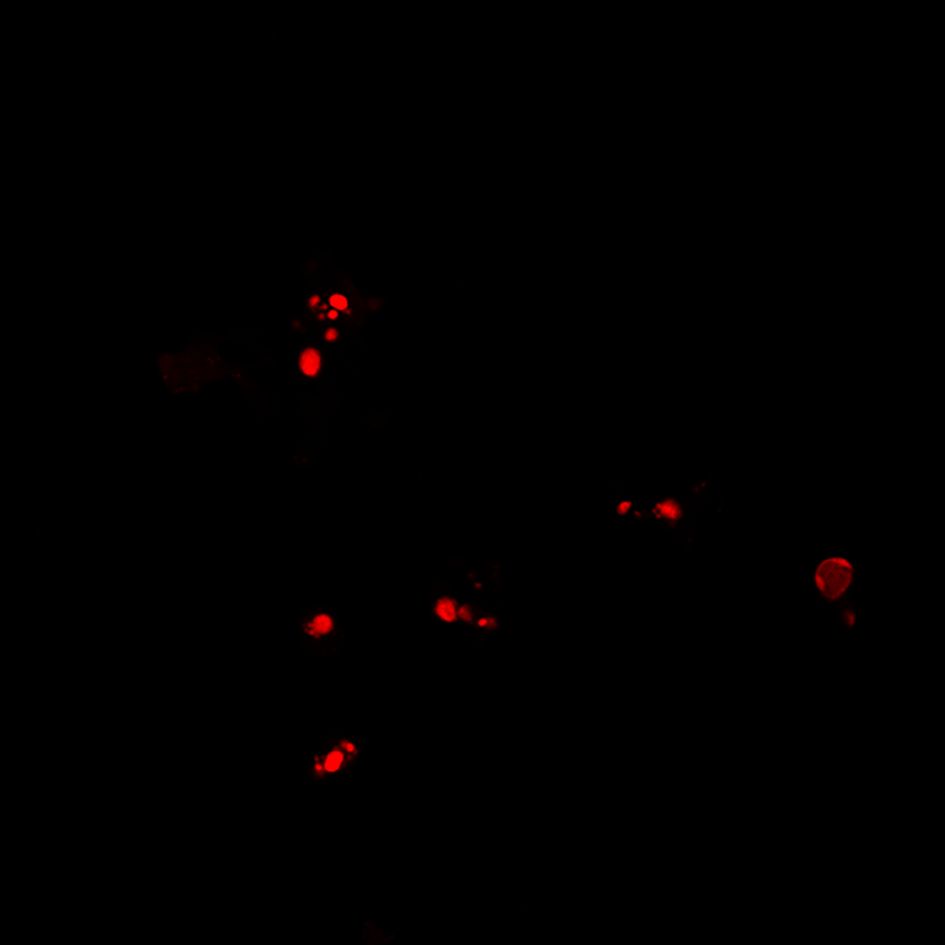

Supplement: Data S1 [file peerj-08-9110-s001.zip › Raw data-Figure 3F.png]

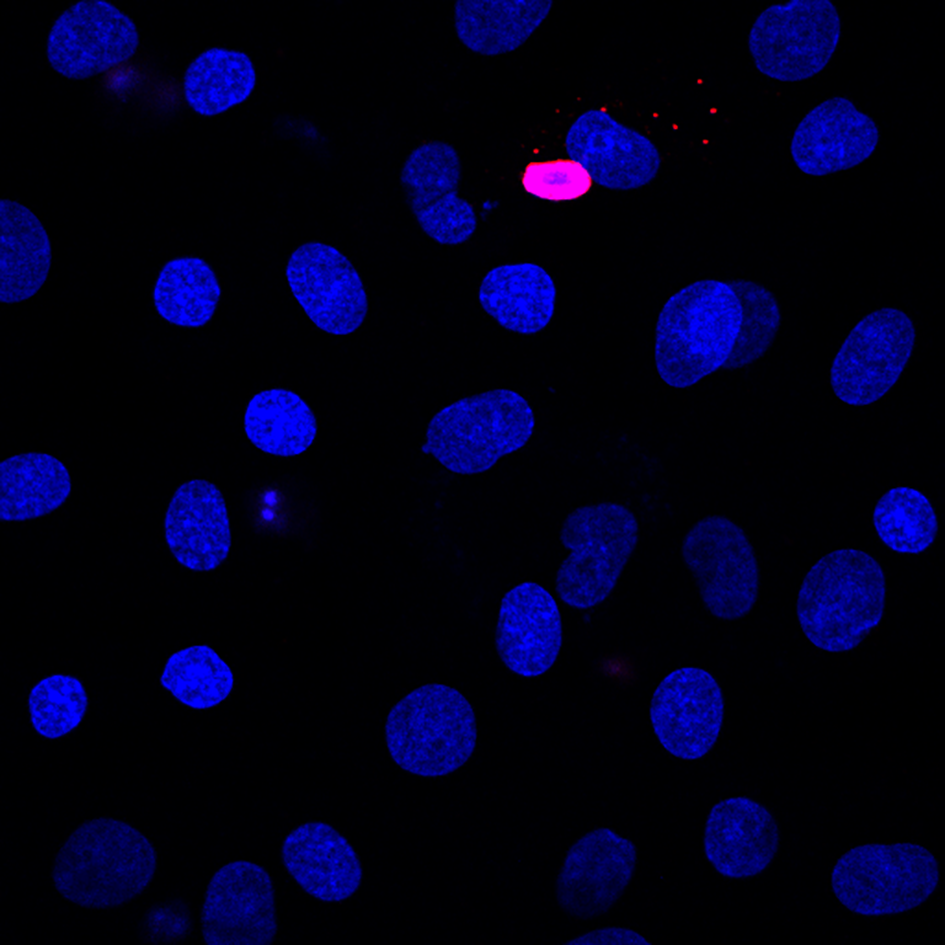

Supplement: Data S1 [file peerj-08-9110-s001.zip › Raw data-Figure 3G.png]

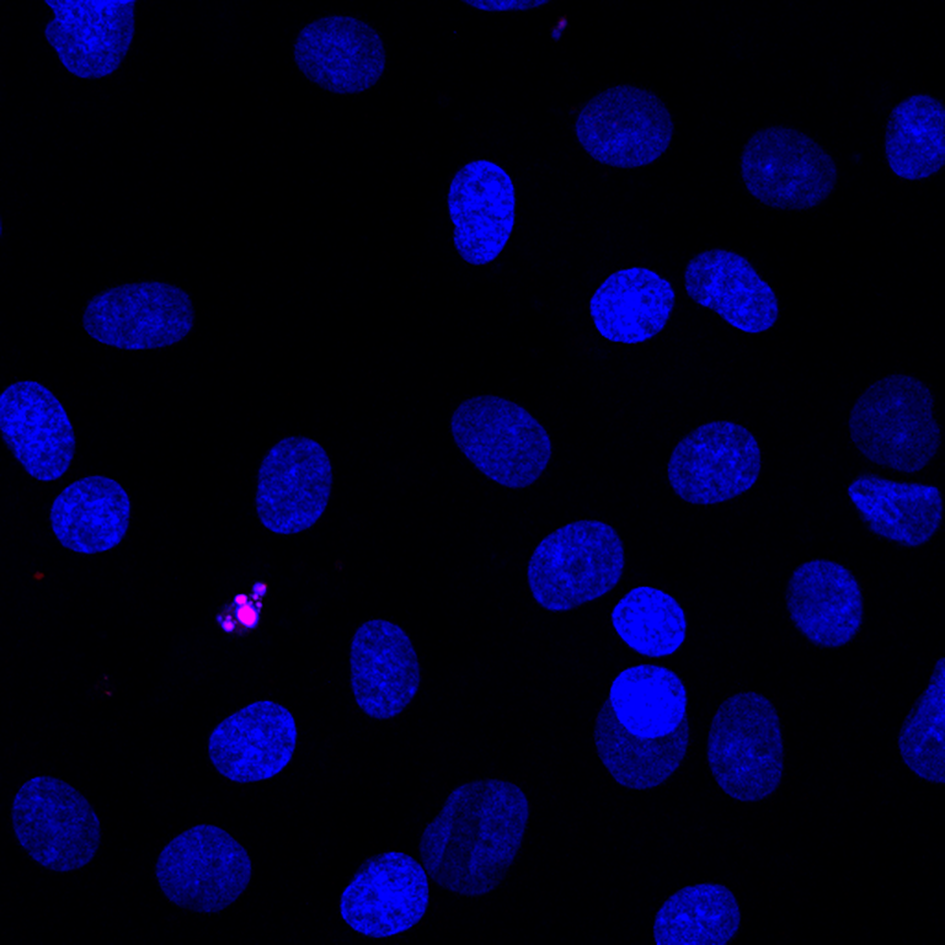

Supplement: Data S1 [file peerj-08-9110-s001.zip › Raw data-Figure 3H.png]

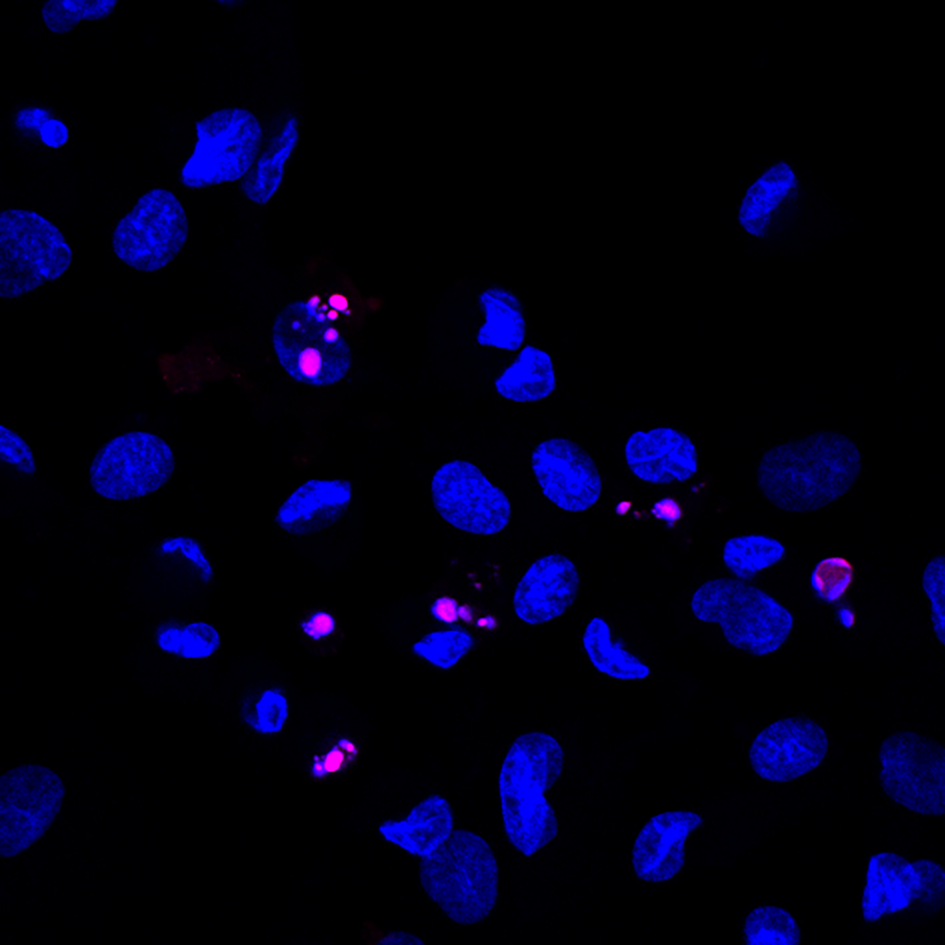

Supplement: Data S1 [file peerj-08-9110-s001.zip › Raw data-Figure 3I.png]

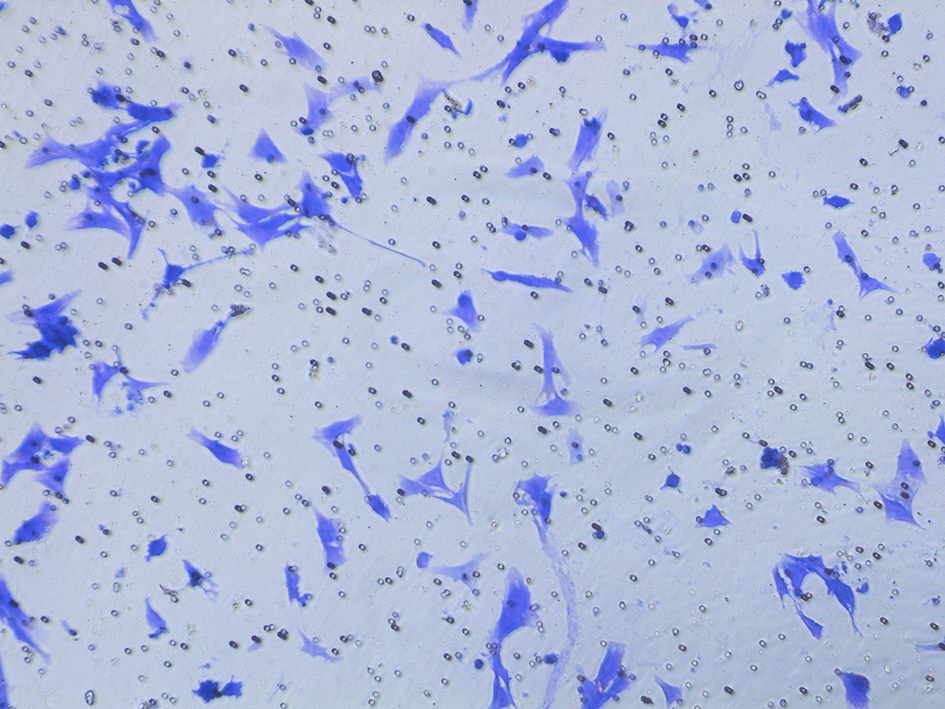

Supplement: Data S1 [file peerj-08-9110-s001.zip › Raw data-Figure 5F.png]

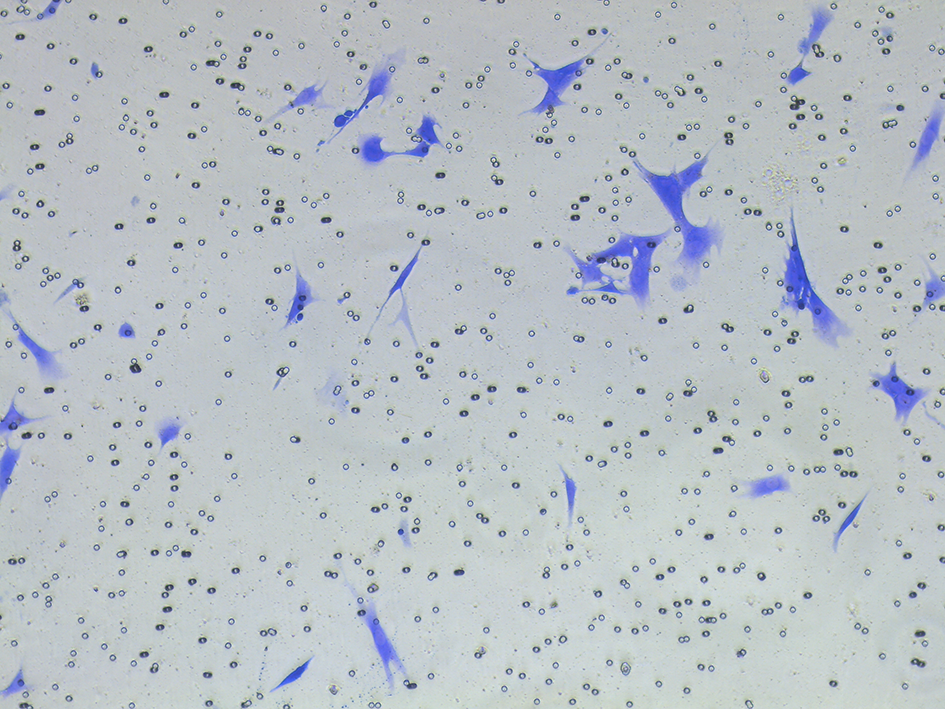

Supplement: Data S1 [file peerj-08-9110-s001.zip › Raw data-Figure 5G.png]

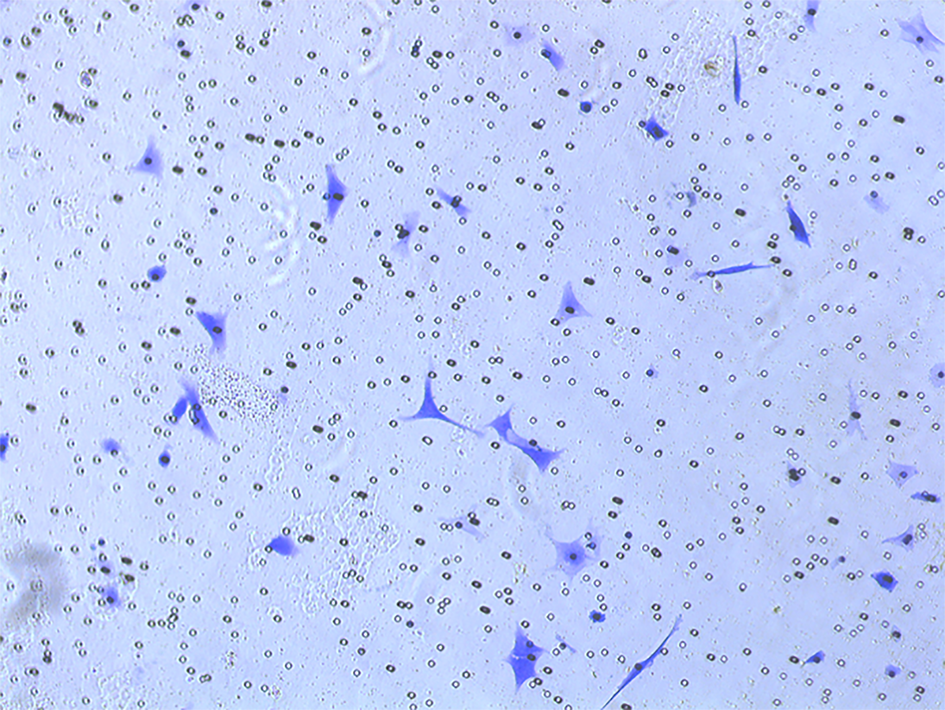

Supplement: Data S1 [file peerj-08-9110-s001.zip › Raw data-Figure 5H.png]

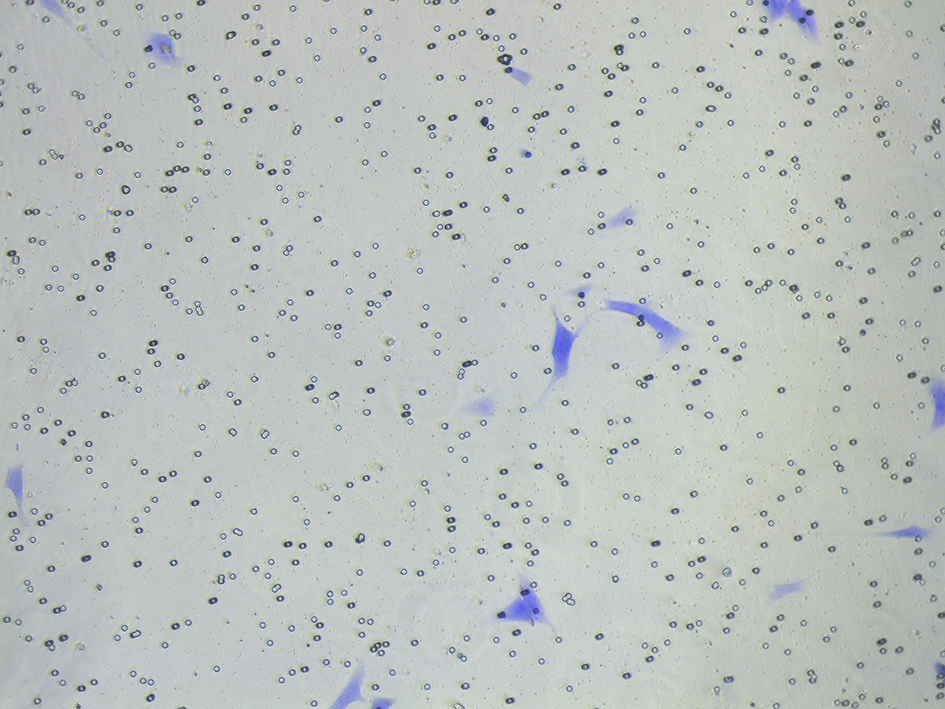

Supplement: Data S1 [file peerj-08-9110-s001.zip › Raw data-Figure 5I.png]

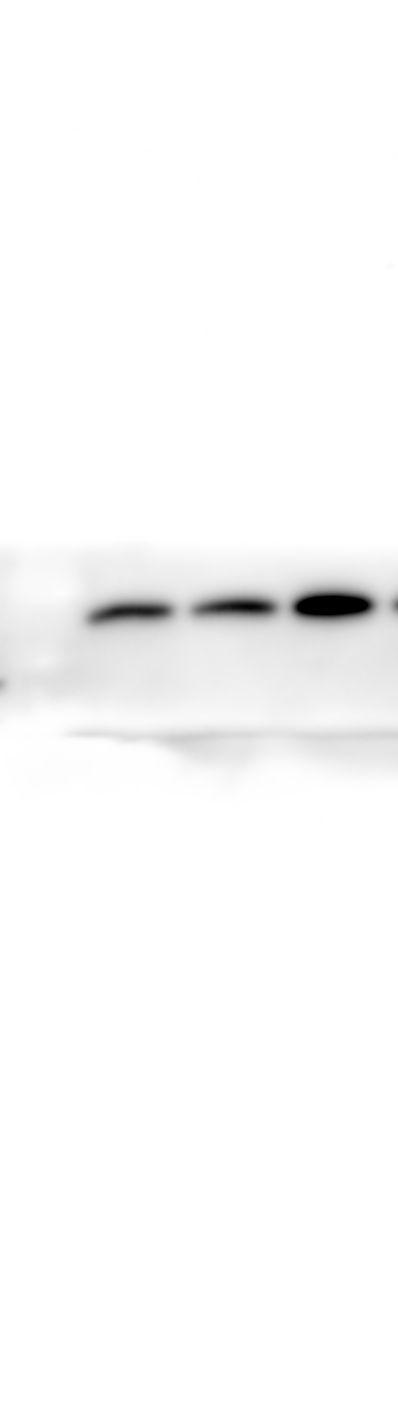

Supplement: Data S1 [file peerj-08-9110-s001.zip › Raw data-WB-Figure 3M.Bax.png]

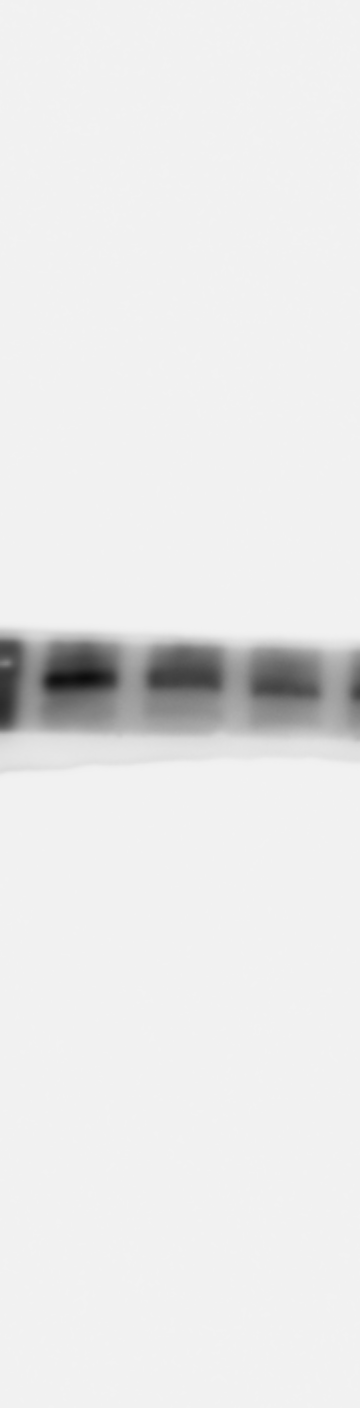

Supplement: Data S1 [file peerj-08-9110-s001.zip › Raw data-WB-Figure 3M.Bcl-2.png]

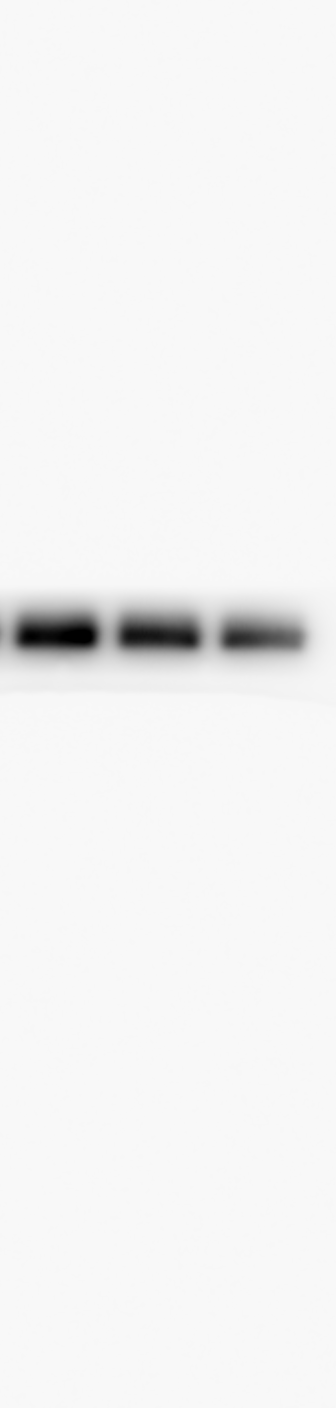

Supplement: Data S1 [file peerj-08-9110-s001.zip › Raw data-WB-Figure 3M.Bcl-xl.png]

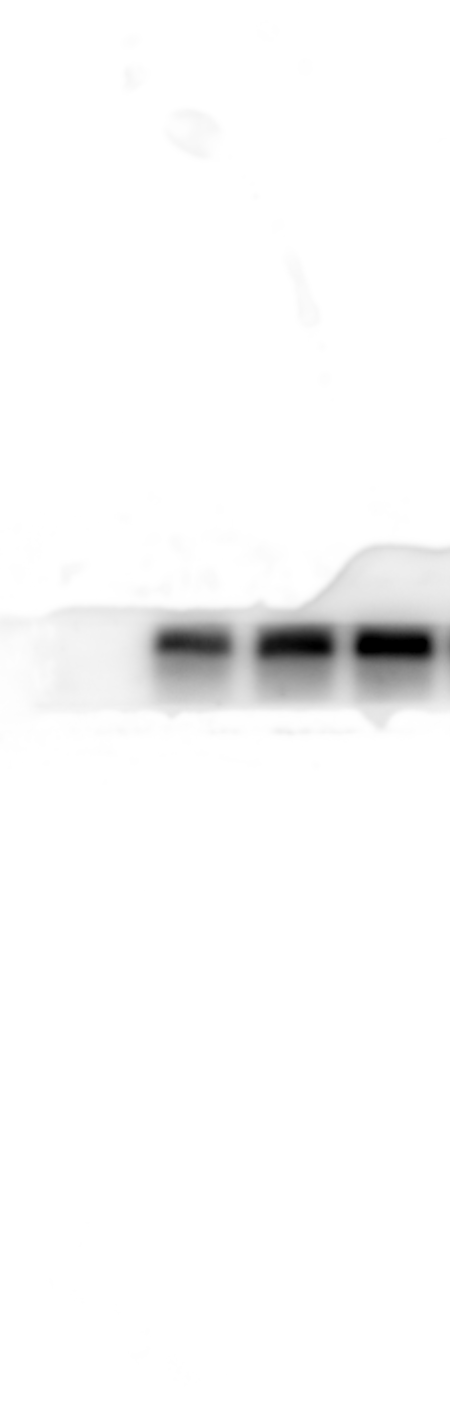

Supplement: Data S1 [file peerj-08-9110-s001.zip › Raw data-WB-Figure 3M.Cleaved caspase-9.png]

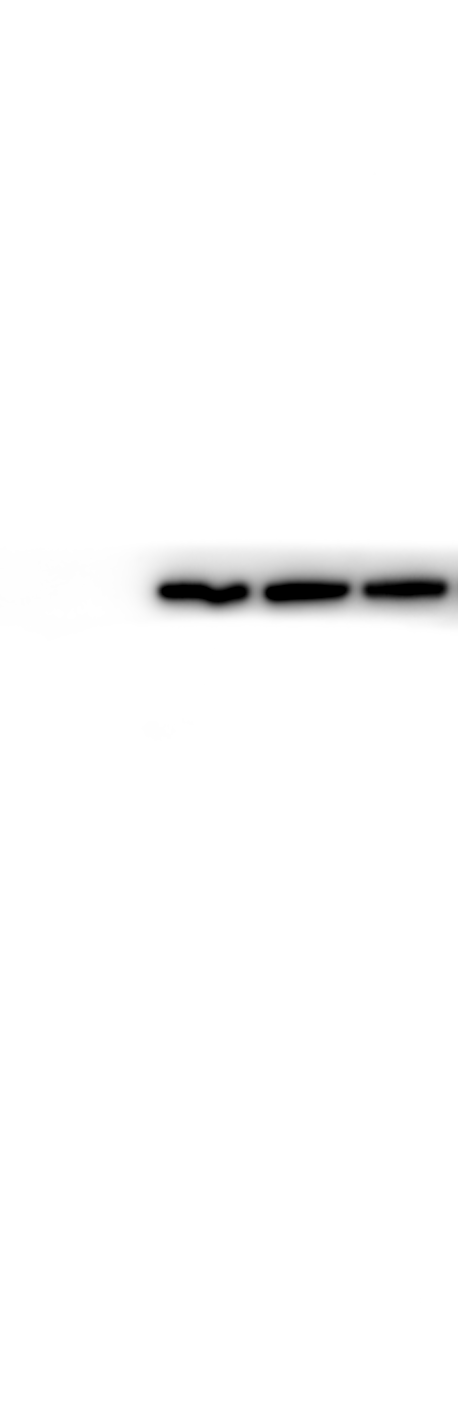

Supplement: Data S1 [file peerj-08-9110-s001.zip › Raw data-WB-Figure 3M.GAPDH.png]

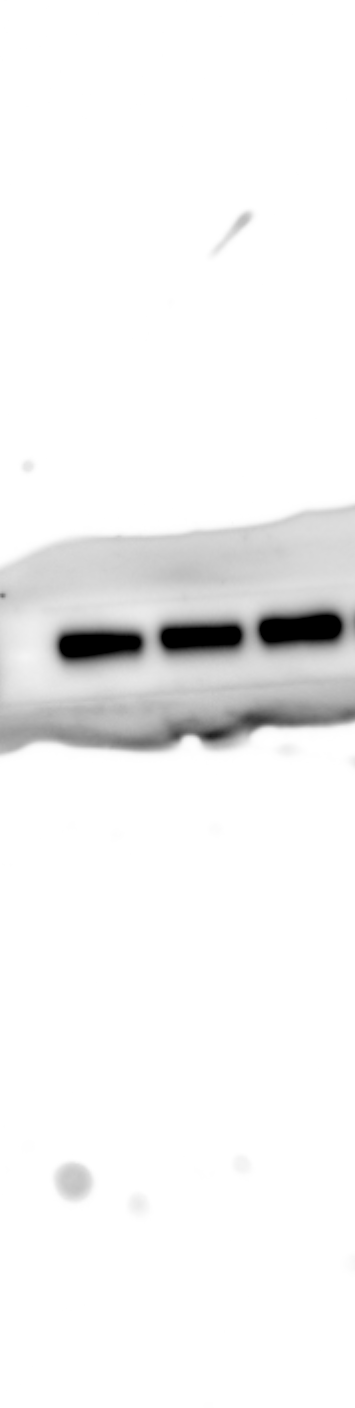

Supplement: Data S1 [file peerj-08-9110-s001.zip › Raw data-WB-Figure 4A.Akt.png]

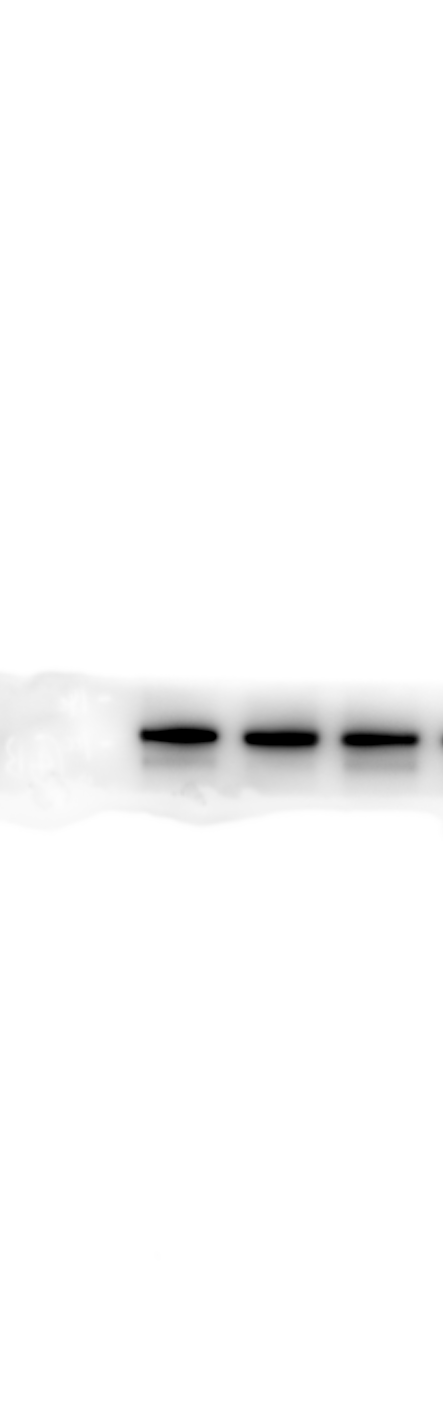

Supplement: Data S1 [file peerj-08-9110-s001.zip › Raw data-WB-Figure 4A.FoxO3a.png]

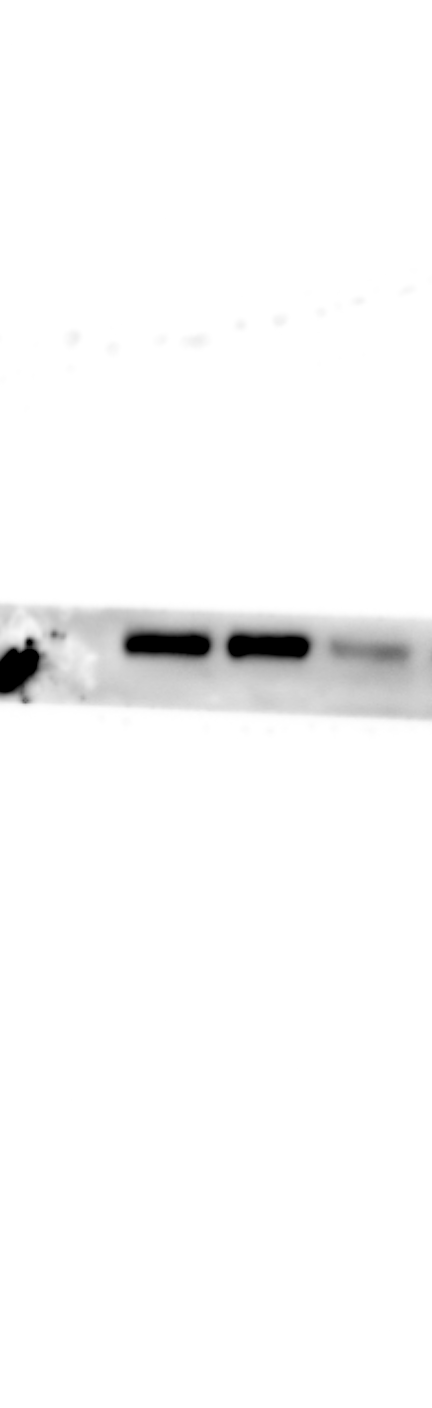

Supplement: Data S1 [file peerj-08-9110-s001.zip › Raw data-WB-Figure 4A.p-Akt.png]

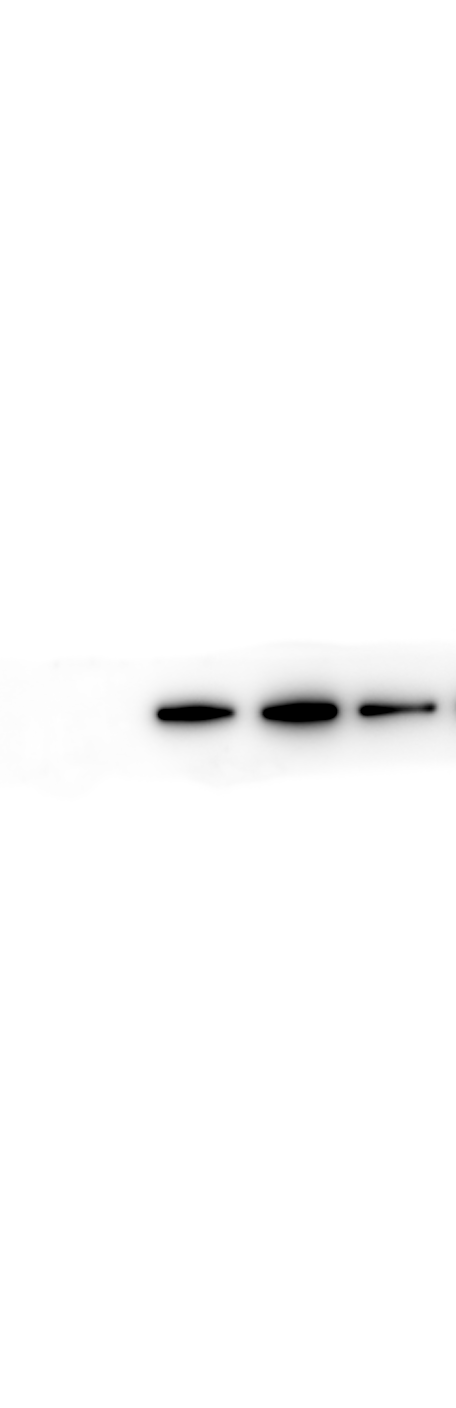

Supplement: Data S1 [file peerj-08-9110-s001.zip › Raw data-WB-Figure 4A.p-FoxO3a .png]

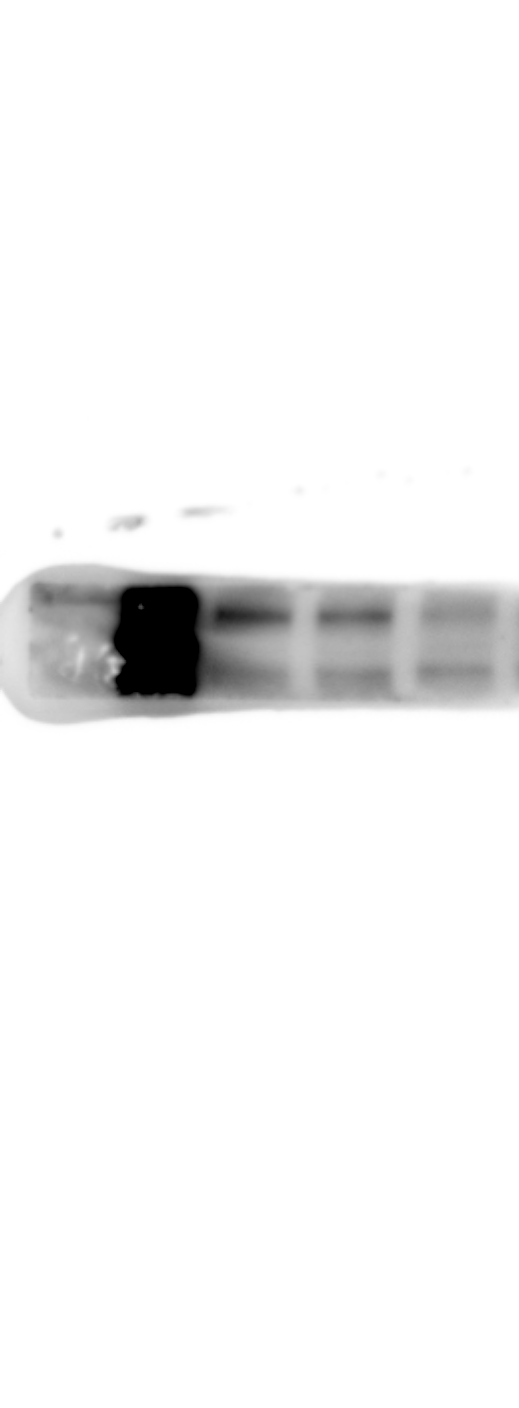

Supplement: Data S1 [file peerj-08-9110-s001.zip › Raw data-WB-Figure 4A.p-PI3K.png]

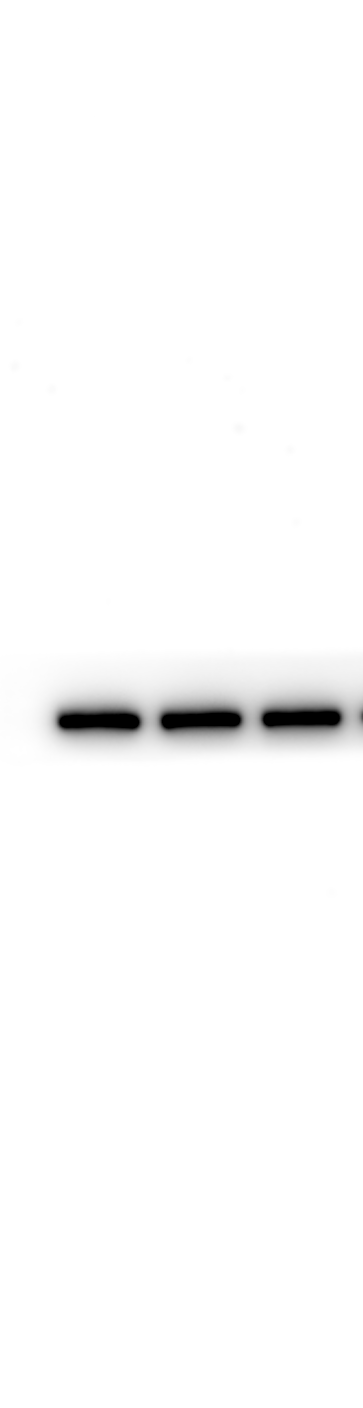

Supplement: Data S1 [file peerj-08-9110-s001.zip › Raw data-WB-Figure 4A.PI3K.png]

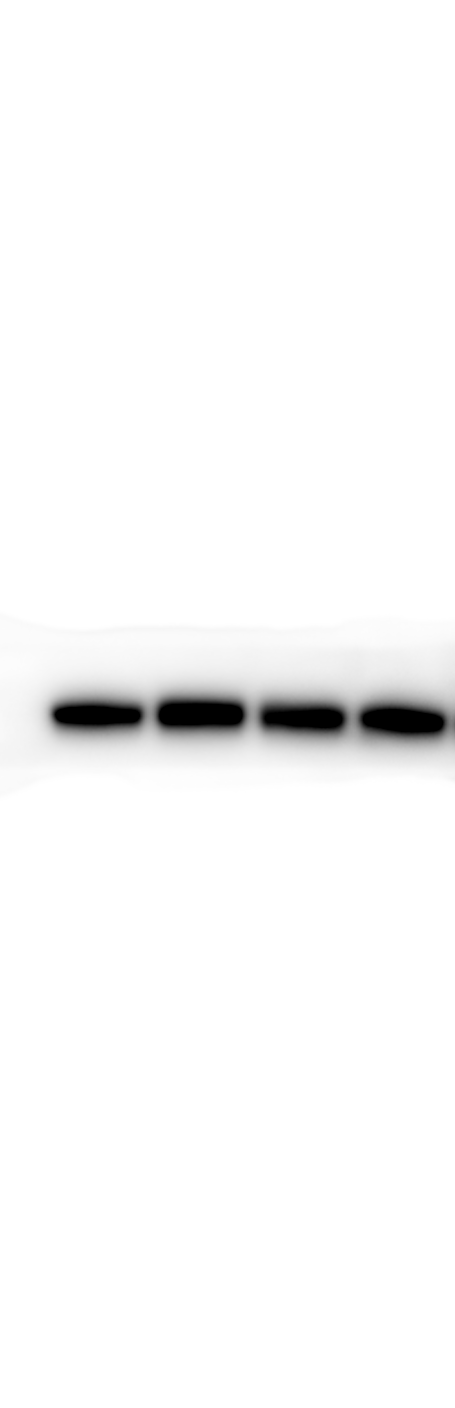

Supplement: Data S1 [file peerj-08-9110-s001.zip › Raw data-WB-Figure 5A.Akt.png]

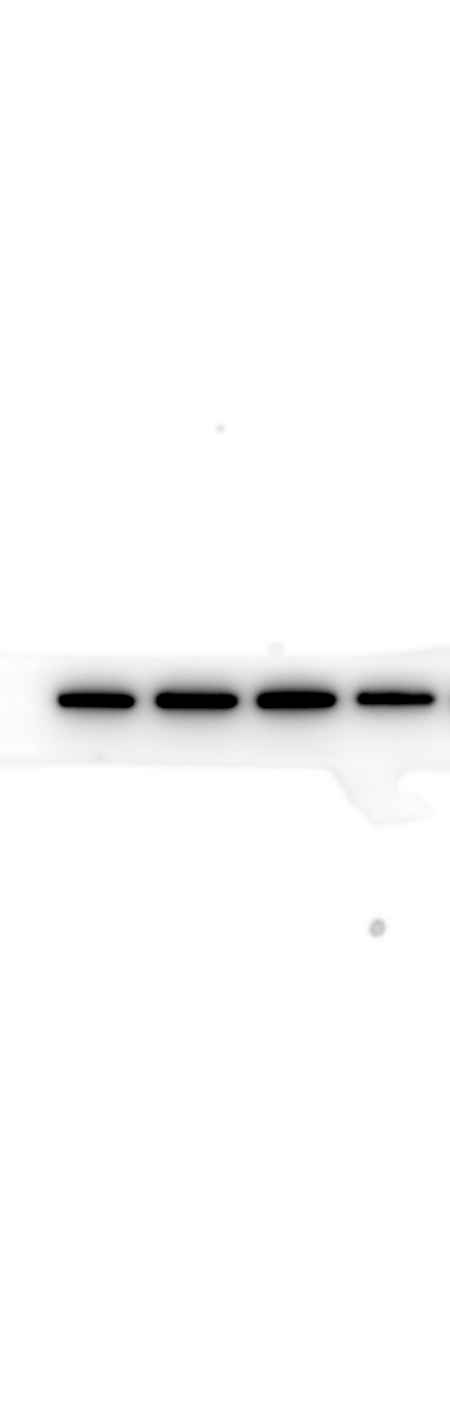

Supplement: Data S1 [file peerj-08-9110-s001.zip › Raw data-WB-Figure 5A.FoxO3a.png]

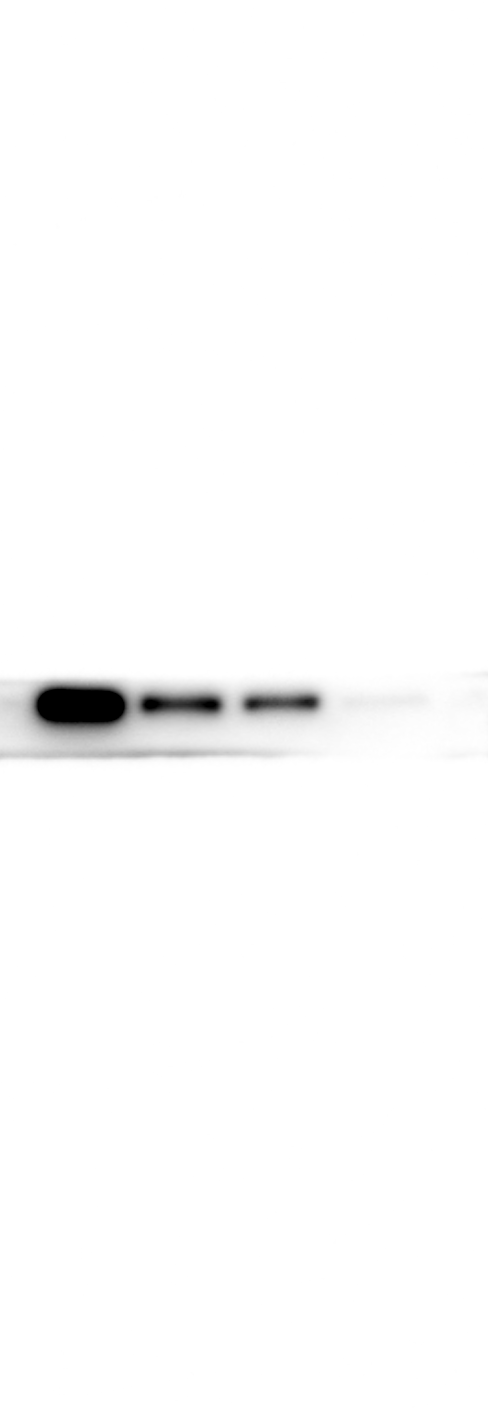

Supplement: Data S1 [file peerj-08-9110-s001.zip › Raw data-WB-Figure 5A.p-Akt.png]

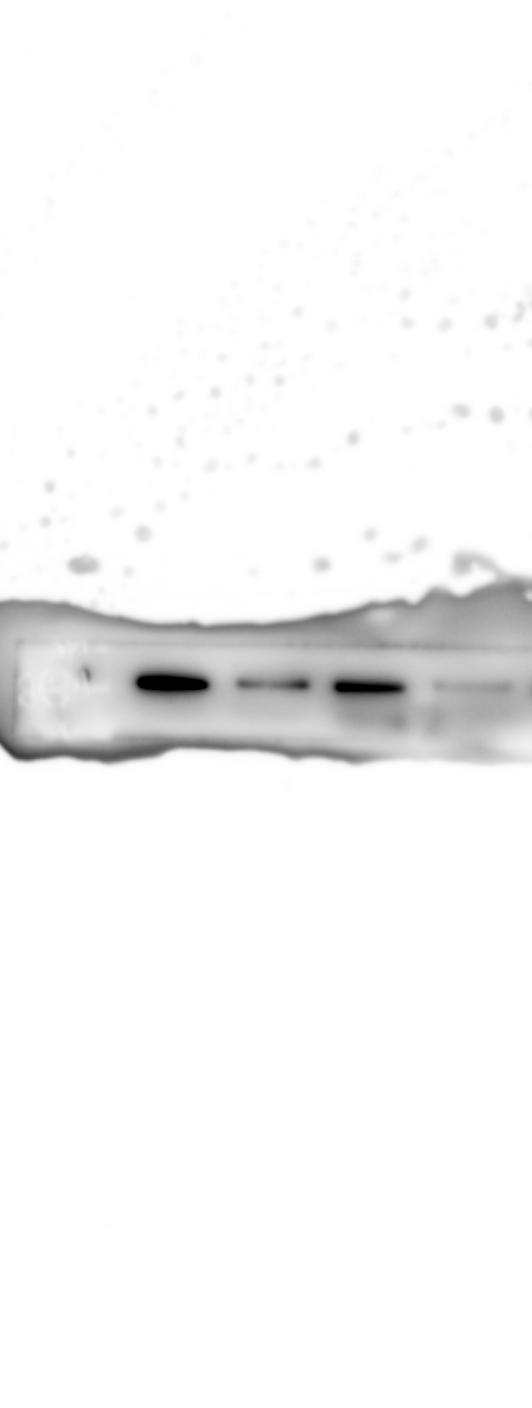

Supplement: Data S1 [file peerj-08-9110-s001.zip › Raw data-WB-Figure 5A.p-FoxO3a.png]

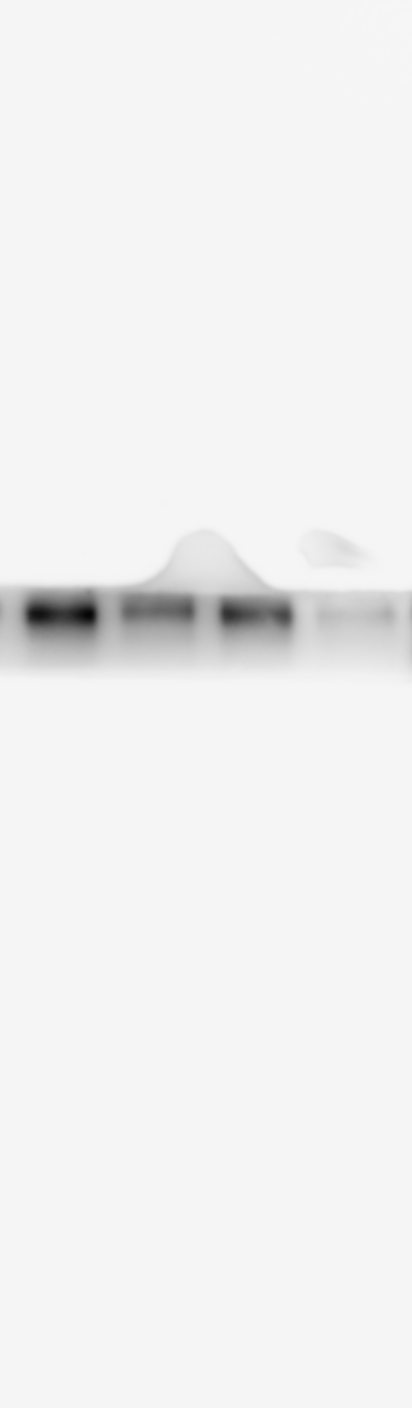

Supplement: Data S1 [file peerj-08-9110-s001.zip › Raw data-WB-Figure 5A.p-PI3K.png]

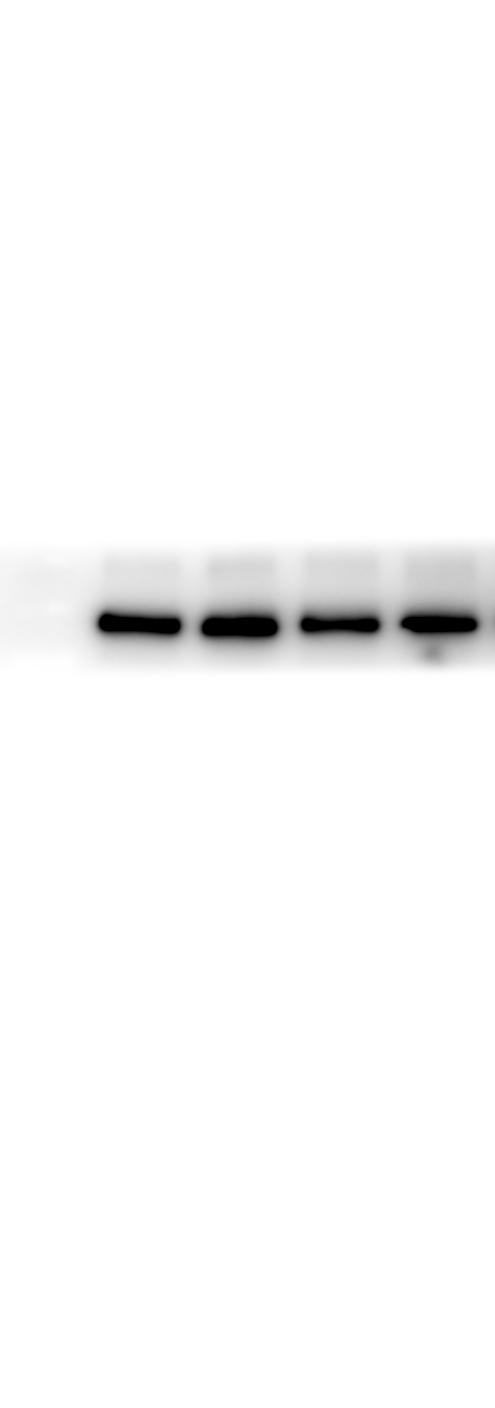

Supplement: Data S1 [file peerj-08-9110-s001.zip › Raw data-WB-Figure 5A.PI3K.png]

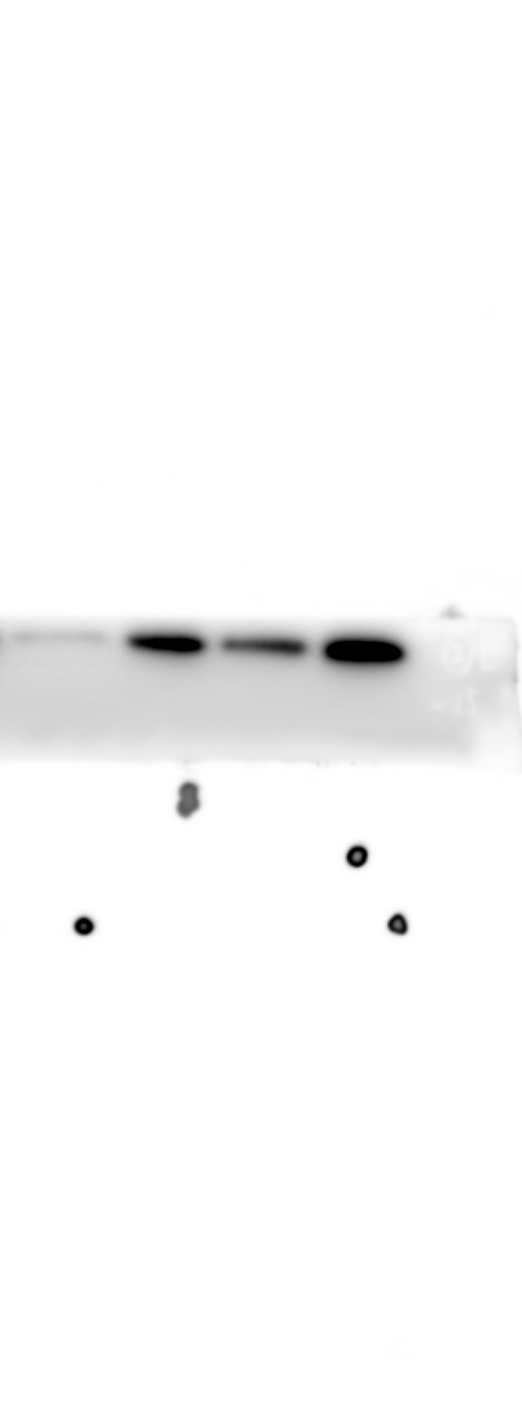

Supplement: Data S1 [file peerj-08-9110-s001.zip › Raw data-WB-Figure 6C.Bax.png]

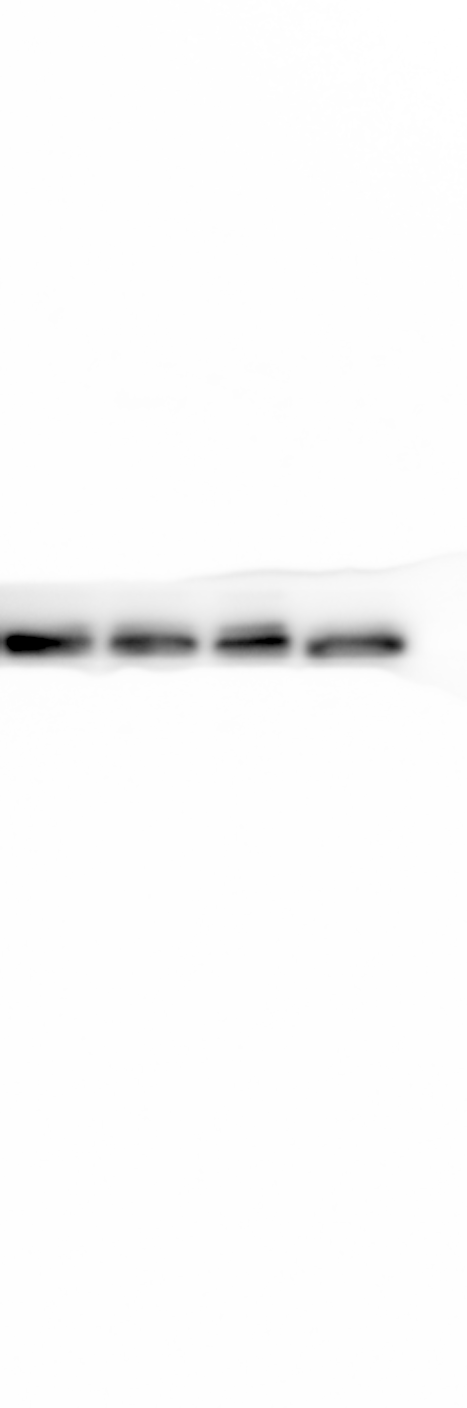

Supplement: Data S1 [file peerj-08-9110-s001.zip › Raw data-WB-Figure 6C.Bcl-2.png]

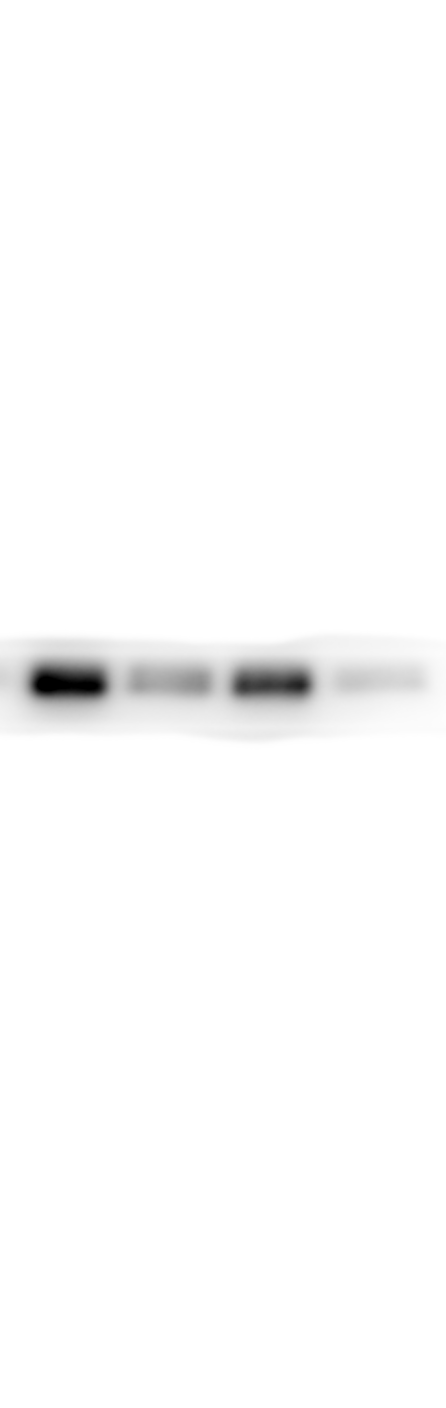

Supplement: Data S1 [file peerj-08-9110-s001.zip › Raw data-WB-Figure 6C.Bcl-xl.png]

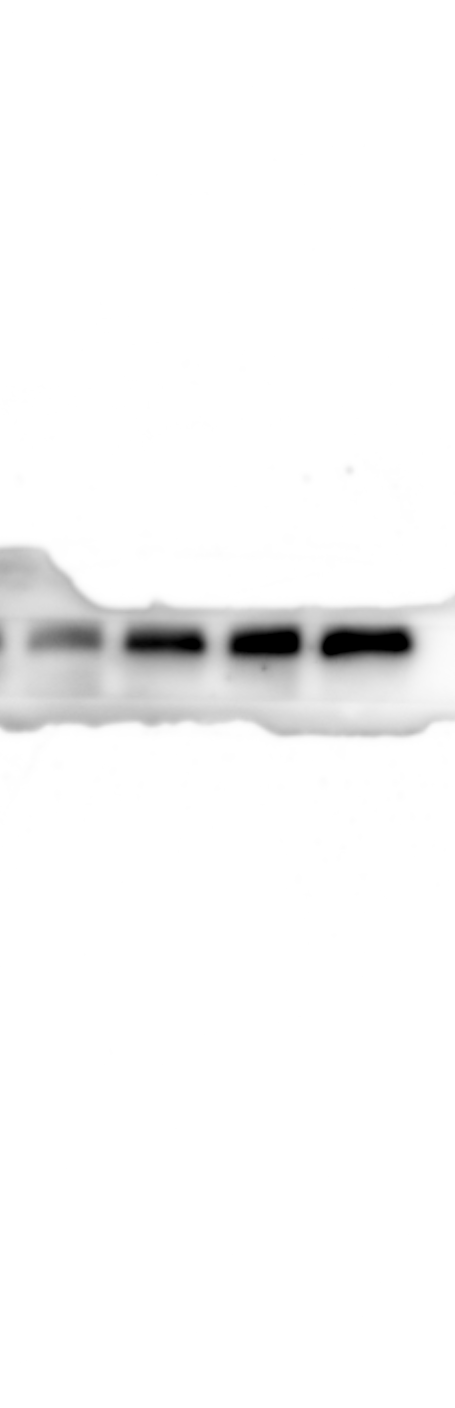

Supplement: Data S1 [file peerj-08-9110-s001.zip › Raw data-WB-Figure 6C.Cleaved caspase-9.png]

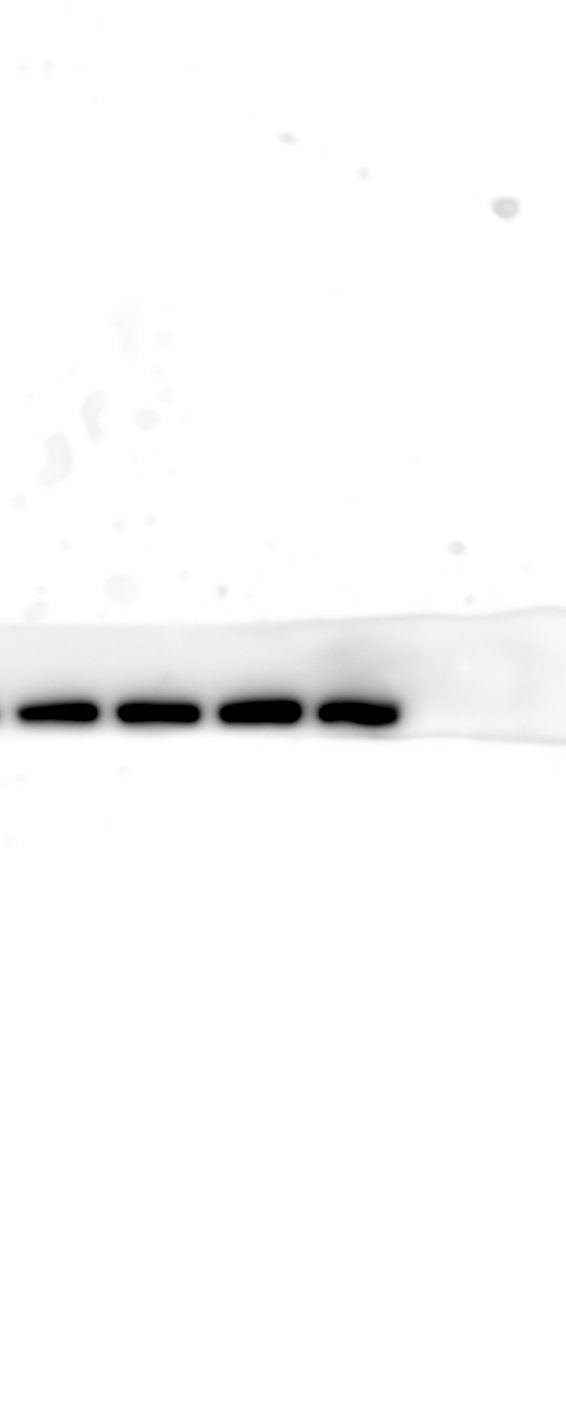

Supplement: Data S1 [file peerj-08-9110-s001.zip › Raw data-WB-Figure 6C.GAPDH.png]
